# MicroRNA‐21a‐5p Promotes Cerebral Angiogenesis in Transient Ischemic Attack by Targeting RBMS3 and Subsequently Modulating the TGFBR1/SMAD2/3 Pathway

**DOI:** 10.1111/cns.70573

**Published:** 2025-08-18

**Authors:** Jiahui Wang, Yanyan Li, Jingyi Wang, Shiling Chen, Luwei Nie, Xuan Wu, Jiarui Li, Ping Zhang, Zhouping Tang

**Affiliations:** ^1^ Department of Neurology, Tongji Hospital, Tongji Medical College Huazhong University of Science and Technology Wuhan China; ^2^ Department of Neurology, the Affiliated Changsha Central Hospital, Hengyang Medical School University of South China Changsha China

**Keywords:** angiogenesis, miRNA‐21a‐5p, RBMS3, TGFBR1, transient ischemic attack

## Abstract

**Aims:**

Transient ischemic attack (TIA) serves as a crucial precursor to a potential stroke. Angiogenesis is essential for the renovation of the damaged brain after TIA. This study aimed to elucidate the role of miRNA‐21a‐5p in angiogenesis following TIA and unravel the underlying mechanisms.

**Methods:**

In vivo and in vitro TIA models were established using the modified suture middle cerebral artery occlusion and oxygen–glucose deprivation/reoxygenation methods. Differentially expressed miRNAs in the TIA group versus the control group were identified using small RNA sequencing. The putative target genes of miRNA‐21a‐5p were predicted via bioinformatics analysis and validated using a dual luciferase reporter gene assay. TIA models were then treated with miRNA‐21a‐5p antagomir/agomir or/and an adeno‐associated virus interfering with the target gene to assess the effects of miRNA‐21a‐5p and the identified target gene on angiogenesis after TIA.

**Results:**

MiRNA‐21a‐5p was the miRNA with the most significant changes after TIA. RBMS3 was identified as a target gene for miRNA‐21a‐5p. Downregulation of miRNA‐21a‐5p expression reduced angiogenesis in animal and cellular models of TIA, and miRNA‐21a‐5p upregulation had a completely opposite effect. RBMS3 suppression reversed miRNA‐21a‐5p knockdown‐mediated inhibition of angiogenesis in TIA models. Moreover, the TGFBR1/SMAD2/3 pathway was found to be downstream for miRNA**‐**21a‐5p/RBMS3.

**Conclusion:**

MiRNA‐21a‐5p conferred neuroprotective effects against TIA by enhancing angiogenesis through the RBMS3/TGFBR1/SMAD2/3 pathway.

## Graphical abstract

A schematic diagram showing the modulatory effect of miRNA‐21a‐5p on angiogenesis after TIA. The miRNA‐21a‐5p levels were elevated after TIA. MiRNA‐21a‐5p inhibited the expression of the target gene RBMS3 by binding to its 3′UTR site, and then activated the TGFBR1/SMAD2/3 signaling pathway, thereby enhancing angiogenesis.

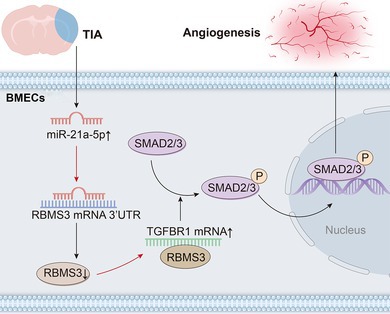

## 1 Introduction

Transient ischemic attack (TIA) refers to a transient neurological impairment caused by focal cerebral, spinal, or retinal ischemia, without acute cerebral infarction [1]. However, the absence of cerebral infarct lesions on magnetic resonance imaging (MRI) does not imply a lack of brain tissue injury. A few animal studies and our previous study have reported that TIA mice exhibited selective neuronal apoptosis and necrosis [2, 3, 4, 5]. There is also growing evidence that TIA serves as a crucial precursor to stroke [6] and is related to a higher risk of long‐term functional disability or death [7]. Moreover, TIA patients may experience affective disorders, cognitive decline, brain atrophy, and other long‐term adverse prognostic events [8, 9, 10, 11], which reflect persistent brain tissue damage at a subclinical level. TIA has been shown to be an independent risk factor for increased brain atrophy [12, 13]. Cerebral blood flow disorders and regional hypoperfusion after TIA may trigger neuroinflammation and breakdown of the blood–brain barrier, contributing to progressive accumulation of small vessel diseases and neurodegeneration [14, 15], and then brain atrophy [16]. The whole‐brain atrophy rates parallel the progression of pre‐clinical and clinical cognitive decline [13]. Recent studies have found that the onset of TIA can lead to functional impairment in the cingulate gyrus and superior longitudinal fasciculus in the white matter region, thus causing cognitive decline [10, 17]. In addition, an ischemic lesion located in the corona radiata may interfere with the functional circuitry between the brainstem and frontal cortex, thereby affecting the emotional expression of patients [17]. Nevertheless, so far, studies on TIA have mainly focused on secondary prevention, with less attention paid to renovation after brain injury. Hence, there is an urgent need for novel targets in the treatment of TIA.

Angiogenesis is essential to the recovery of damaged brain tissue after TIA; thus, it may provide an effective therapeutic strategy for TIA. MicroRNAs (miRNAs) are a class of small non‐coding RNA molecules that participate in post‐transcriptional modulation of gene expression by binding to the 3′ untranslated region (3′UTR) of target mRNAs [18, 19]. The dysfunction of miRNAs has been identified in multiple diseases, including ischemic stroke [20, 21, 22]. However, changes in miRNA expression after TIA remain unknown. MiRNA‐21a‐5p is highly expressed in vascular smooth muscle cells [23] and endothelial cells [24]. In addition, miRNA‐21a‐5p modulates tumor cell proliferation and migration and is closely associated with angiogenesis in cancer and non‐neoplastic diseases [25, 26, 27, 28, 29]. Nevertheless, the effect of miRNA‐21a‐5p on angiogenesis after TIA has scarcely been investigated.

RNA binding motif single stranded interacting protein 3 (RBMS3) gene encodes an RNA‐binding protein that mainly regulates RNA metabolism, cell cycle, and apoptosis [30]. RBMS3 is a vital inhibitor of breast cancer cell proliferation and invasion [31], as well as nasopharyngeal carcinoma microvessel formation [32]. The transforming growth factor beta (TGF‐β) signaling pathway plays an essential role in angiogenesis by modulating endothelial cell stability, ependymal cell function, and the production and release of pro‐angiogenic factors [33, 34, 35]. Interestingly, a study has revealed that RBMS3 could regulate chondrogenesis and craniofacial development via the TGF‐β pathway [36].

We previously developed a TIA model in C57BL/6 mice through 8‐min occlusion of one middle cerebral artery (MCA), without permanent neurological deficits or cerebral infarcts [5]. In the present study, this rodent model was employed to mimic human TIA. Moreover, the cellular TIA model was established by modifying the exposure duration of oxygen–glucose deprivation/reoxygenation (OGD/R) in primary cultured mouse brain microvascular endothelial cells (mBMECs). Herein, we aimed to elucidate the role of miRNA‐21a‐5p in angiogenesis after TIA and unravel the underlying mechanisms.

## 2 Materials and Methods

The Data S1 include an expanded “Supplementary Materials and Methods” (Tables S1 and S2). The original image of Western blot are shown in the Data S2.

### 2.1 Animals

Adult male C57BL/6 mice (8–10 weeks) were obtained from the Hubei Research Center for Laboratory Animals (Wuhan, China). The mice were raised in a standard environment at 20°C–26°C under a 12‐h light/dark cycle, with unlimited access to food and water. The study protocol was approved by the Experimental Animal Ethics Committee of Tongji Hospital, Tongji Medical College, Huazhong University of Science and Technology. All experimental groups were randomized, and outcomes were analyzed by independent investigators blinded to the experimental conditions.

### 2.2 Establishment of Mouse TIA Model

As described in our previous work [5], a mouse TIA model was established by occluding the origin of the right MCA for 8 min with a suture, based on the following criteria: (1) objective evidence of ischemia and reperfusion, as determined by cerebral blood flow (CBF) measurement during middle cerebral artery occlusion (MCAO) surgery; (2) no permanent neurological impairment, as assessed by the Zea‐Longa 5‐point score scale at 24 h after the onset of reperfusion; and (3) no acute cerebral infarction, as detected by MRI examination (including T2WI and diffusion weighted imaging (DWI) sequences) at 24 h after reperfusion. If the three criteria were met, the TIA model was considered successfully established (Figure S1).

### 2.3 Intracerebroventricular Administration of miRNA

As described previously [37, 38], miRNA‐21a‐5p antagomir and negative control (NC) antagomir (RiboBio, China) were diluted with normal saline to a final concentration of 1 μM, and miRNA‐21a‐5p agomir and NC agomir (RiboBio, China) were diluted to 0.5 μM for intracerebroventricular injection. The mice underwent isoflurane anesthesia, and their heads were secured on a stereotaxic frame. Subsequently, 2 μL of diluted miRNA solution was injected into the contralateral cerebral ventricle of mice (bregma 0: 1 mm left lateral, 0.5 mm posterior, and 2.6 mm deep) using a nanoliter microsyringe (WPI, USA). The injection rate was 0.2 μL/min, and to prevent liquid reflux, the microsyringe was allowed to stabilize for an additional 15 min before slowly withdrawing.

### 2.4 Stereotaxic Injection of Adeno‐Associated Virus

Adeno‐associated virus (AAV)‐RBMS3 short hairpin RNA‐GFP (AAV‐shRBMS3; 5.98 × 10^13^ viral genomes/mL; the target sequence: 5′‐TCCCACAGACATCTATCAC‐3′) and AAV‐control (3.45 × 10^13^ viral genomes/mL) were prepared by Weizhen Biotechnology Co. Ltd. (Shandong, China). AAV9 was used as the serotype. Twenty‐one days before TIA modeling, AAV‐shRBMS3 or AAV‐control was stereotaxically injected into the striatum and cortex (0.6 μL/site at a rate of 0.12 μL/min) using the coordinates referenced from the bregma as follows: 2.3 mm right lateral, 0.4 mm anterior, and depths of 1.8 and 3.5 mm. The microsyringe was left at the injection site for another 15 min and then withdrawn slowly.

### 2.5 Primary mBMECs Culture and OGD/R Treatment

Neonatal C57BL/6 mice (7–10 days of age) were provided by BIONT Biotechnology Co. Ltd. (Wuhan, China) for the extraction of primary mBMECs as described previously [39]. Oxygen–glucose deprivation (OGD) combined with reoxygenation was employed to simulate TIA in vitro. For OGD, mBMECs (90% confluency) were washed and incubated with deoxygenated glucose‐free DMEM (Procell, China), followed by transferring to a hypoxic incubator filled with 1% O_2_/94% N_2_/5% CO_2_ at 37°C for a specific duration. For reoxygenation, the medium was renewed with a standard culture medium, and then cells were restored to normal incubation conditions. Control cells were cultured in standard medium under normoxic conditions (37°C, 95% air/5% CO_2_) without OGD/R treatment.

### 2.6 Cell Transfection

MiRNA‐21a‐5p inhibitor (5′‐UCAACAUCAGUCUGAUAAGCUA‐3′), RBMS3 small interfering RNA (si‐RBMS3; the target sequence: 5′‐TCCCACAGACATCTATCAC‐3′), and their negative controls were constructed by RiboBio Biotechnology Co. Ltd. (Guangzhou, China). mBMECs were seeded into 6‐well plates 1 day prior to transfection. Then, 150 nM miRNA‐21a‐5p inhibitor or NC inhibitor and 75 nM si‐RBMS3 or si‐NC were transfected into mBMECs (70%–80% confluency) with lipofectamine 3000 (Invitrogen, USA) 36 h before OGD.

### 2.7 Statistical Analysis

Statistical tests were conducted using GraphPad Prism v9.0. The normality of data was assessed using the Shapiro–Wilk test. Data with normal distribution were shown as mean ± standard deviation (SD). An unpaired two‐tailed *t*‐test was utilized for comparisons between two groups, and one‐way analysis of variance (one‐way ANOVA) with Tukey's post hoc test was applied for multiple‐group comparisons. A *p*‐value < 0.05 was deemed statistically significant.

## 3 Results

### 3.1 MiRNA‐21a‐5p Was Up‐Regulated in Brain Tissues Following TIA

**FIGURE 1:**
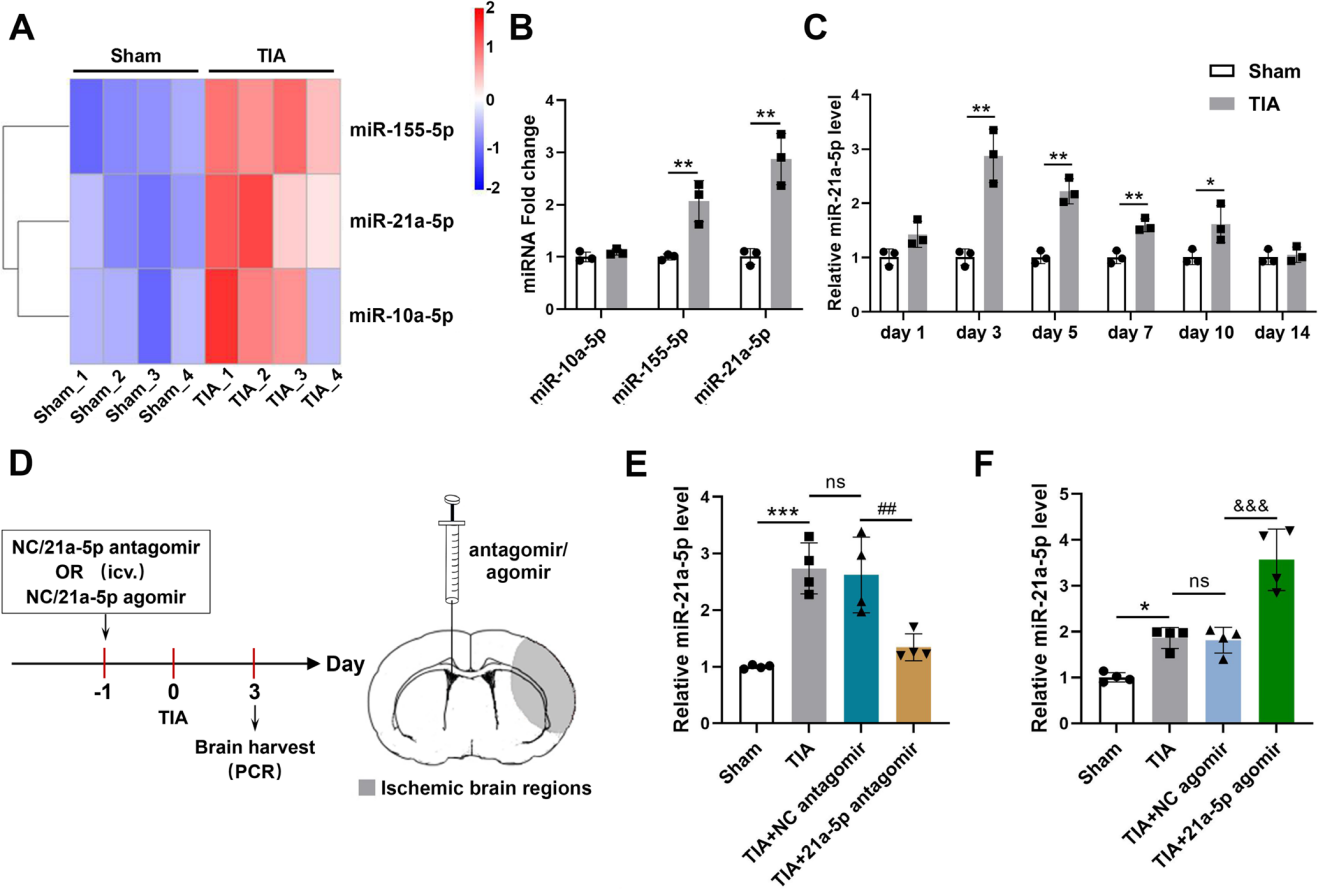
MiRNA‐21a‐5p was up‐regulated in the ischemic brain tissues of mouse TIA model. (A) The clustering heatmap of differentially expressed miRNAs in TIA mice versus sham mice quantified by small RNA sequencing. (B) The mRNA levels of the three miRNAs identified by RT‐qPCR (*n* = 3). (C) RT‐qPCR analysis of miRNA‐21a‐5p expression in mouse brains at different time points after TIA (*n* = 3). (D) The timeline of miRNA injection, TIA modeling, and brain tissue collection. A schematic diagram revealing the injection position of antagomir or agomir (contralateral cerebral ventricle) and the areas of brain sampling (the gray zone, equal to the ischemic region in the MCAO procedure). (E) RT‐qPCR analysis of miRNA‐21a‐5p level in TIA mice after injecting miRNA‐21a‐5p antagomir or NC antagomir (*n* = 4). (F) RT‐qPCR analysis of miRNA‐21a‐5p level in TIA mice after administering miRNA‐21a‐5p agomir or NC agomir (*n* = 4). Data were presented as mean ± SD. In (B and C), *p < 0.05, **p < 0.01 versus sham group by unpaired two‐tailed *t*‐test. In (E and F), *p < 0.05, ***p < 0.001 versus sham group; ns, no significance versus TIA group; ^##^ *p* < 0.01 versus TIA + NC antagomir group; ^&&&^ *p* < 0.001 versus TIA + NC agomir group by one‐way ANOVA with Tukey's post hoc test.

To explore the contribution of miRNAs after TIA, we performed small RNA sequencing on the brain tissues of sham‐operated mice and TIA mice. Considering the criteria of fold change > 2 and *Q* value < 0.05, three differentially expressed miRNAs were identified between the two groups and were demonstrated in a volcano plot (Figure S2) and clustering heatmap (Figure 1A), including miRNA‐155‐5p, miRNA‐21a‐5p, and miRNA‐10a‐5p. We also performed RT‐qPCR validation on the differentially expressed miRNAs, and observed that the expression levels of miRNA‐155‐5p and miRNA‐21a‐5p were remarkably higher in the TIA group than those in the sham group (Figure 1B). Thus, the miRNA showing the most significant up‐regulation, namely miRNA‐21a‐5p, was selected as a candidate for further investigation.

Subsequently, the expression of miRNA‐21a‐5p in the ischemic brain tissues was detected at several time points after TIA. Compared with the sham group, miRNA‐21a‐5p levels were markedly up‐regulated, peaking on the 3rd day after TIA, and then gradually returning to the baseline within 14 days (Figure 1C). To investigate the role of miRNA‐21a‐5p in TIA, miRNA‐21a‐5p antagomir or miRNA‐21a‐5p agomir was injected into the cerebral ventricle before TIA (Figure 1D). The RT‐qPCR data revealed that miRNA‐21a‐5p antagomir remarkably decreased the expression of miRNA‐21a‐5p in the brain tissues after TIA (Figure 1E), while miRNA‐21a‐5p agomir markedly increased miRNA‐21a‐5p levels (Figure 1F).

### 3.2 MiRNA‐21a‐5p Promoted Angiogenesis in the Brain Tissues of TIA Mice

**FIGURE 2:**
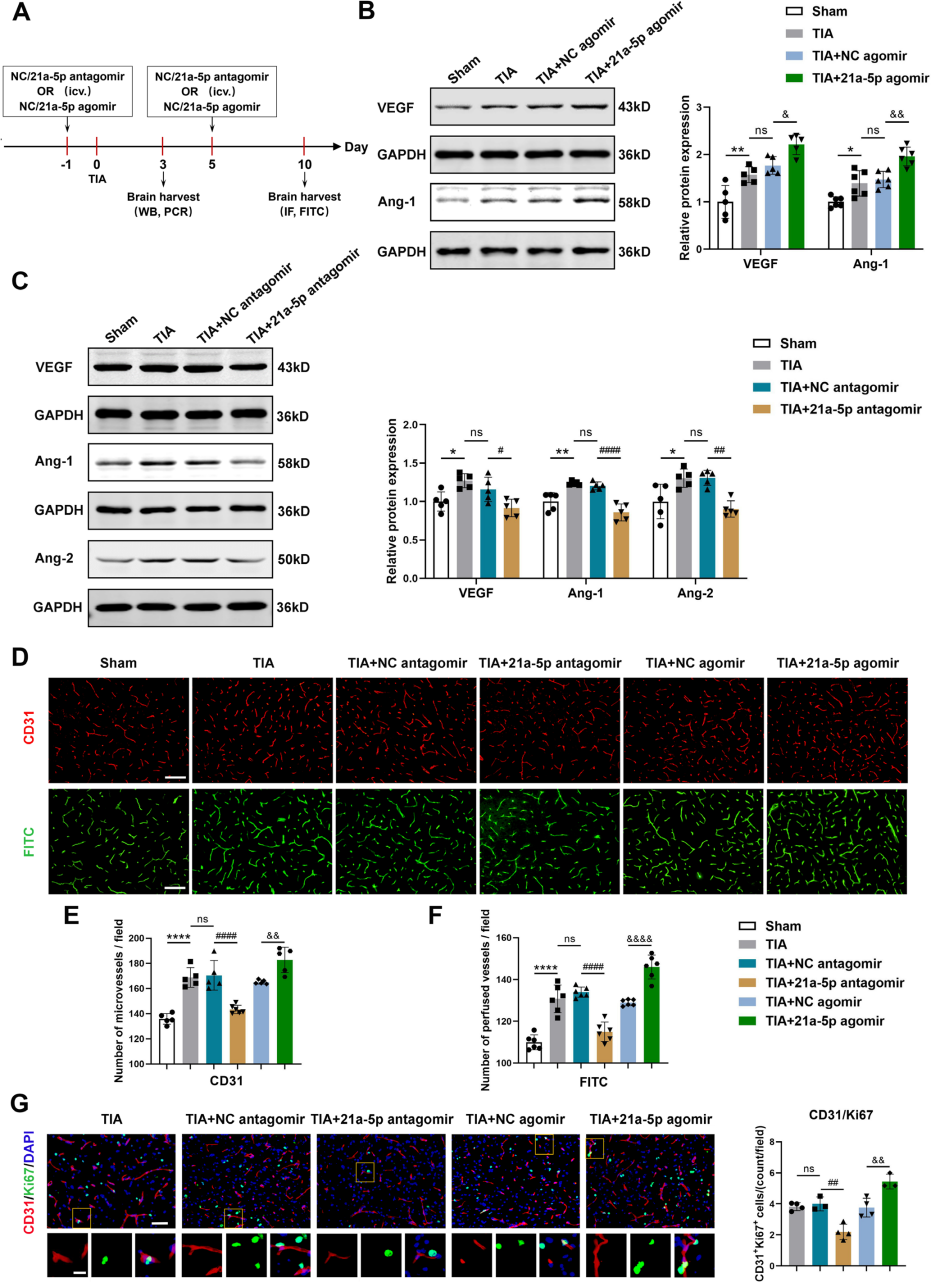
MiRNA‐21a‐5p promoted angiogenesis in brain tissues after TIA. (A) The timeline of miRNA injection, TIA modeling, and brain tissue collection. (B) Representative Western blotting images of VEGF and Ang‐1 in the ischemic brain tissues of the sham, TIA, TIA + NC agomir and TIA + 21a‐5p agomir groups. Bar graphs displaying data quantification (*n* = 5–6). (C) Representative Western blotting images and protein quantitative analysis of VEGF, Ang‐1 and Ang‐2 in the brain tissues of the sham, TIA, TIA + NC antagomir and TIA + 21a‐5p antagomir groups (*n* = 5). (D) Representative images of CD31 immunofluorescent staining and FITC perfusion imaging in the ischemic hemisphere, scale bar = 100 μm. (E) Quantification of CD31‐positive microvessels (*n* = 5–6). (F) Quantitative analysis of FITC‐labeled perfused microvessels (*n* = 6). (G) Representative images showing CD31 (red) and Ki67 (green) double‐immunostaining in the ischemic hemisphere, scale bar = 50 μm. Magnified local views were shown, scale bar = 15 μm. Bar graphs showing the quantification of CD31 and Ki67 double‐positive cells (*n* = 3–4). Data were presented as mean ± SD. *p < 0.05, **p < 0.01, ****p < 0.0001 versus sham group; ns, no significance versus TIA group; ^#^ *p* < 0.05, ^##^ *p* < 0.01, ^####^ *p* < 0.0001 versus TIA + NC antagomir gruop; ^&^ *p* < 0.05, ^&&^ *p* < 0.01, ^&&&&^ *p* < 0.0001 versus TIA + NC agomir group by one‐way ANOVA with Tukey's post hoc test.

The dynamic expression of miRNA‐21a‐5p after TIA was consistent with the time points of angiogenesis after cerebral ischemia reported in previous studies [40, 41]. In addition, miRNA‐21a‐5p was shown to be highly expressed in endothelial cells [42]. Therefore, this study explored the role of miRNA‐21a‐5p in angiogenesis after TIA. 24 h before and 5 days after TIA modeling, mice were contralaterally given intracerebroventricular injection of NC antagomir, miRNA‐21a‐5p antagomir, NC agomir, or miRNA‐21a‐5p agomir. Mice were then sacrificed at 3 and 10 days after TIA (Figure 2A). The expression levels of some angiogenic growth factors in the brain were determined by Western blotting 3 days after TIA. Our results indicated that the protein levels of VEGF and Ang‐1 were markedly elevated in the TIA + 21a‐5p agomir group compared to the TIA + NC agomir group (Figure 2B). Conversely, compared to the TIA + NC antagomir group, the protein levels of VEGF, Ang‐1, and Ang‐2 were markedly decreased in the TIA + 21a‐5p antagomir group (Figure 2C).

Next, we conducted CD31 immunofluorescent staining and fluorescein isothiocyanate (FITC) perfusion imaging to determine the density of microvessels and perfused microvessels in the ischemic hemisphere 10 days after TIA, respectively. It was found that the density of CD31‐positive microvessels and FITC‐labeled perfused microvessels was remarkably decreased in the TIA + 21a‐5p antagomir group compared to the TIA + NC antagomir group. Conversely, the density of CD31‐positive microvessels and FITC‐labeled perfused microvessels in the TIA + 21a‐5p agomir group was obviously increased compared to the TIA + NC agomir group. The microvessel density exhibited no remarkable difference between the TIA, TIA + NC antagomir, and TIA + NC agomir groups (Figure 2D–F). To further determine the impact of miRNA‐21a‐5p on newly formed microvessels, CD31 and Ki67 double immunofluorescent staining was performed. We found that the number of CD31/Ki67 double‐positive cells in the ischemic hemisphere of the TIA + 21a‐5p antagomir group was markedly reduced compared with the TIA + NC antagomir group. On the contrary, the number of CD31/Ki67 double‐positive cells was significantly higher in the TIA + 21a‐5p agomir group than that in the TIA + NC agomir group (Figure 2G). Taken together, these findings demonstrated that upregulation or downregulation of miRNA‐21a‐5p could enhance or inhibit angiogenesis in the brains of TIA mice, respectively.

### 3.3 RBMS3 Was a Direct Target of miRNA‐21a‐5p

**FIGURE 3:**
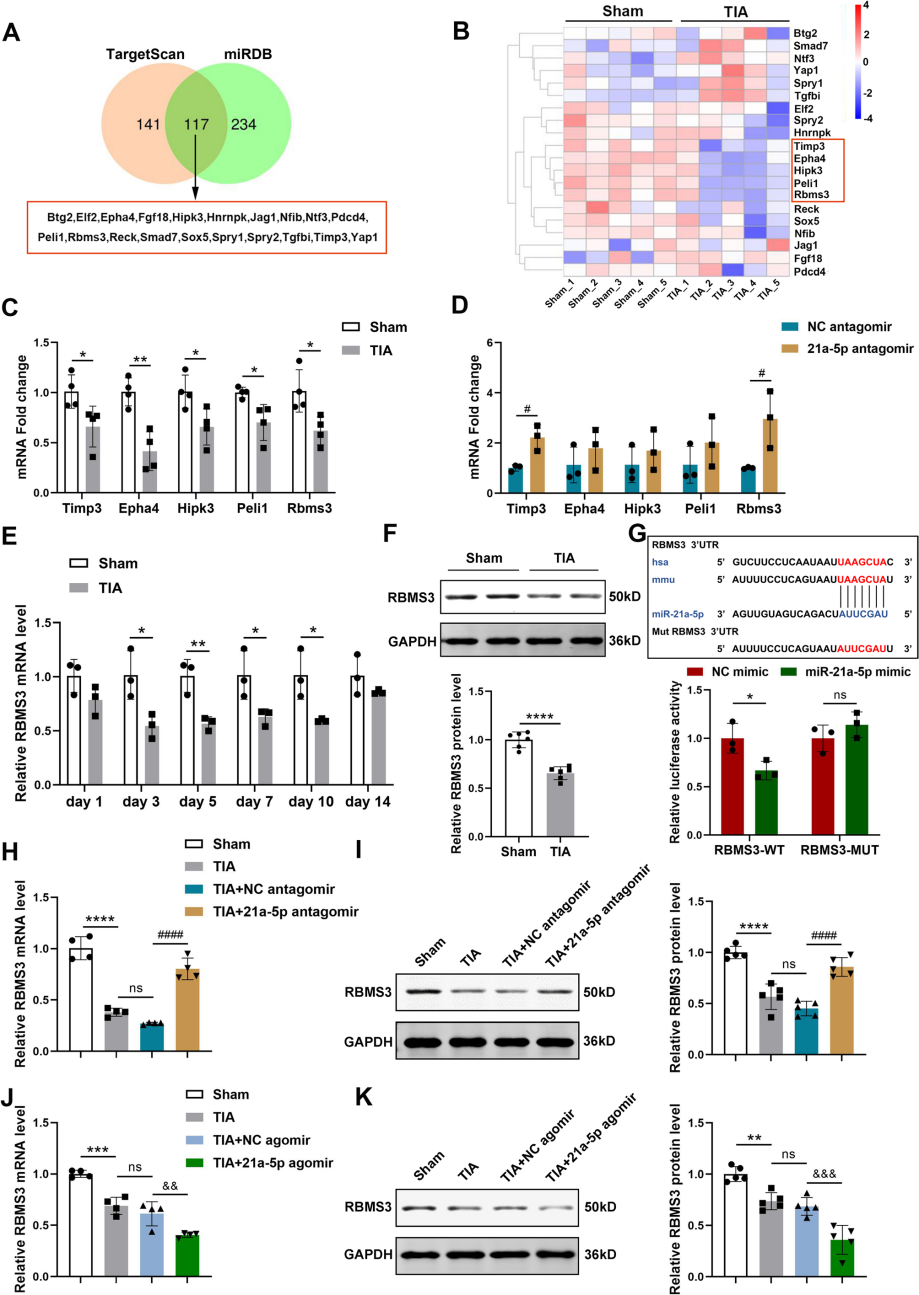
RBMS3 was the direct target for miRNA‐21a‐5p. (A) A Venn diagram illustrating the overlapping target genes of miRNA‐21a‐5p predicted by TargetScan and miRDB databases, with 20 candidate genes selected for further analysis. (B) The clustering heatmap of target genes associated with angiogenesis in TIA mice versus sham mice quantified by transcriptome sequencing. (C) RT‐qPCR analysis for the mRNA levels of the five candidate target genes in the brain tissues of the sham and TIA groups (*n* = 4). (D) RT‐qPCR analysis of the five candidate genes in the NC antagomir group and 21a‐5p antagomir group (*n* = 3). (E) Detection of RBMS3 mRNA levels in mouse brains at various time points after TIA by RT‐qPCR (*n* = 3). (F) Detection of RBMS3 protein levels in the brain 3 days after TIA by Western blotting (*n* = 6). (G) The binding site between miRNA‐21a‐5p and 3′UTR of RBMS3. Bar graphs showing the relative luciferase activity in cultured BEND3 co‐transfected with corresponding miRNA mimics and luciferase plasmid containing the wild‐type (WT) or mutant‐type (MUT) 3′UTR sites of RBMS3 (*n* = 3). (H, I) Detection of the mRNA and protein levels of RBMS3 in brain tissues after injecting miRNA‐21a‐5p antagomir or NC antagomir by RT‐qPCR (H) and Western blotting (I) (*n* = 4–5). (J, K) Detection of the mRNA and protein levels of RBMS3 in the brain after treatment with miRNA‐21a‐5p agomir or NC agomir by RT‐qPCR (J) and Western blotting (K) (*n* = 4–5). Data were presented as mean ± SD. In (C–F), *p < 0.05, **p < 0.01, ****p < 0.0001 versus sham group; ^#^ *p* < 0.05 versus NC antagomir group by unpaired two‐tailed *t*‐test. In (G), *p < 0.05, ns, no significance versus NC mimic group by unpaired two‐tailed *t*‐test. In (H–K), **p < 0.01, ***p < 0.001, ****p < 0.0001 versus sham group; ns, no significance versus TIA group; ^####^ *p* < 0.0001 versus TIA + NC antagomir group; ^&&^ *p* < 0.01, ^&&&^ *p* < 0.001 versus TIA + NC agomir group by one‐way ANOVA with Tukey's post hoc test.

To investigate the molecular mechanism through which miRNA‐21a‐5p regulated angiogenesis after TIA, the possible target genes of miRNA‐21a‐5p were predicted using the TargetScan and miRDB databases, and 117 overlapping target genes were obtained after the intersection of the two databases. Based on the literature review, 20 candidate genes related to angiogenesis were identified (Figure 3A). Generally, miRNAs negatively modulate the expression of their target genes; thus, we performed transcriptome sequencing for cluster analysis on the 20 screened target genes (Figure 3B, Table S3). The findings demonstrated that, contrary to miRNA‐21a‐5p expression, the expression levels of TIMP3, EPHA4, HIPK3, PELI1, and RBMS3 were down‐regulated after TIA, which was further validated by RT‐qPCR (Figure 3C). Then, it was confirmed that the downregulation of miRNA‐21a‐5p remarkably increased the mRNA levels of TIMP3 and RBMS3 in the brain (Figure 3D). In addition, the mRNA expression of RBMS3 was significantly reduced on Days 3, 5, 7, and 10 after TIA (Figure 3E), and the protein level of RBMS3 also declined on Day 3 after TIA (Figure 3F), which was found to be negatively correlated with miRNA‐21a‐5p expression in the brain tissues of TIA mice.

Subsequently, the target interaction was verified by dual luciferase reporter gene assay in cultured BEND3. MiRNA‐21a‐5p mimic markedly decreased the luciferase activity in cells containing the wild‐type 3′UTR sites of RBMS3, but not in those containing the mutant‐type 3′UTR (Figure 3G). The results confirmed that miRNA‐21a‐5p directly bound to the 3′UTR site of RBMS3. Furthermore, we observed that the mRNA and protein levels of RBMS3 were markedly higher in the TIA + 21a‐5p antagomir group than those in the TIA + NC antagomir group (Figure 3H,I). On the contrary, both RBMS3 mRNA and protein levels were remarkably declined in the TIA + 21a‐5p agomir group compared to the TIA + NC agomir group (Figure 3J,K). Additionally, neither NC antagomir nor NC agomir altered RBMS3 expression in TIA mice (Figure 3H–K). Collectively, these findings implied that miRNA‐21a‐5p downregulated the expression level of RBMS3 in brain tissues, confirming that RBMS3 was a target gene for miRNA‐21a‐5p.

### 3.4 RBMS3 Suppression Reversed the Inhibitory Effect of miRNA‐21a‐5p Knockdown on Angiogenesis in the Brain Following TIA

**FIGURE 4:**
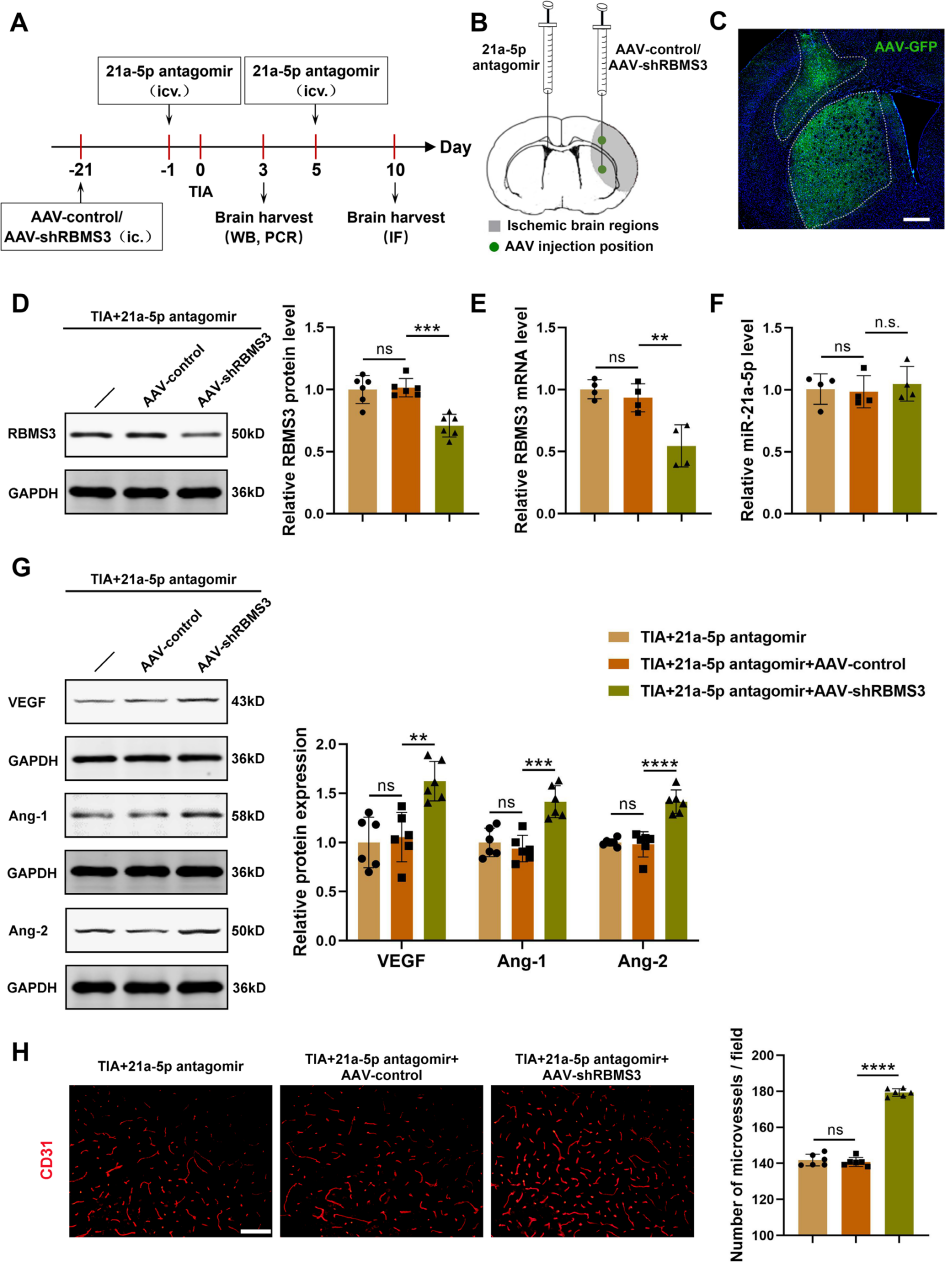
Suppression of RBMS3 reversed the inhibitory effect of miRNA‐21a‐5p knockdown on angiogenesis in TIA mice. (A) The time points of AAV injection, miRNA administration, TIA modeling, and brain tissue acquisition. (B) A schematic diagram revealing the injection positions of AAV (ipsilateral striatum and cortex) and antagomir (contralateral cerebral ventricle), and the areas of brain sampling (the gray area). (C) A representative image of brain sections in mice injected with AAV‐shRBMS3‐GFP (green), scale bar = 400 μm. (D, E) Detection of the protein and mRNA levels of RBMS3 in the brain tissues after different treatments by Western blotting (D) and RT‐qPCR (E) (*n* = 4–6). (F) Detection of the mRNA levels of miRNA‐21a‐5p in the brain by RT‐qPCR (*n* = 4). (G) Representative Western blotting images and protein quantitative analysis of VEGF, Ang‐1 and Ang‐2 in the brain tissues after different treatments (*n* = 6). (H) Representative CD31 immunofluorescent staining images in the ischemic hemisphere, scale bar = 100 μm. Bar graphs displaying the quantitative analysis of CD31‐positive microvessels (*n* = 6). Data were presented as mean ± SD. ns, no significance versus TIA + 21a‐5p antagomir group; **p < 0.01, ***p < 0.001, ****p < 0.0001, n.s., no significance versus TIA + 21a‐5p antagomir + AAV‐control group by one‐way ANOVA with Tukey's post hoc test.

To investigate whether the confirmed target gene RBMS3 was involved in miRNA‐21a‐5p‐mediated regulation of angiogenesis after TIA, a constructed AAV specifically interfering with RBMS3 expression (AAV‐shRBMS3) or AAV‐control was injected into the ipsilateral striatum and cortex 21 days before TIA modeling. In addition, miRNA‐21a‐5p antagomir was injected into the contralateral cerebral ventricle 24 h before and 5 days after TIA. We then sacrificed mice 3 and 10 days after TIA and collected the brain samples (Figure 4A,B). The constructed AAV‐shRBMS3 was verified to be effective in infecting the ipsilateral striatum and adjacent cortex (Figure 4C) and inhibiting the expression of RBMS3 in mouse brains (Figure 4D,E), but did not affect miRNA‐21a‐5p levels (Figure 4F).

Compared with AAV‐control, AAV‐shRBMS3 significantly elevated the protein levels of some angiogenic growth factors in TIA mice injected with miRNA‐21a‐5p antagomir, including VEGF, Ang‐1, and Ang‐2 (Figure 4G). Moreover, it was found that the density of CD31‐positive microvessels was notably elevated in the TIA + 21a‐5p antagomir + AAV‐shRBMS3 group compared to the TIA + 21a‐5p antagomir + AAV‐control group (Figure 4H). Taken together, repression of RBMS3 expression partly abolished the inhibitory effect of miRNA‐21a‐5p knockdown on angiogenesis in TIA‐modeling mice, indicating that miRNA‐21a‐5p regulated angiogenesis by targeting RBMS3 in TIA mice.

### 3.5 The Exposure of Cultured mBMECs to OGD for 1 h Mimicked TIA‐Induced Cell Damage in Vitro

**FIGURE 5:**
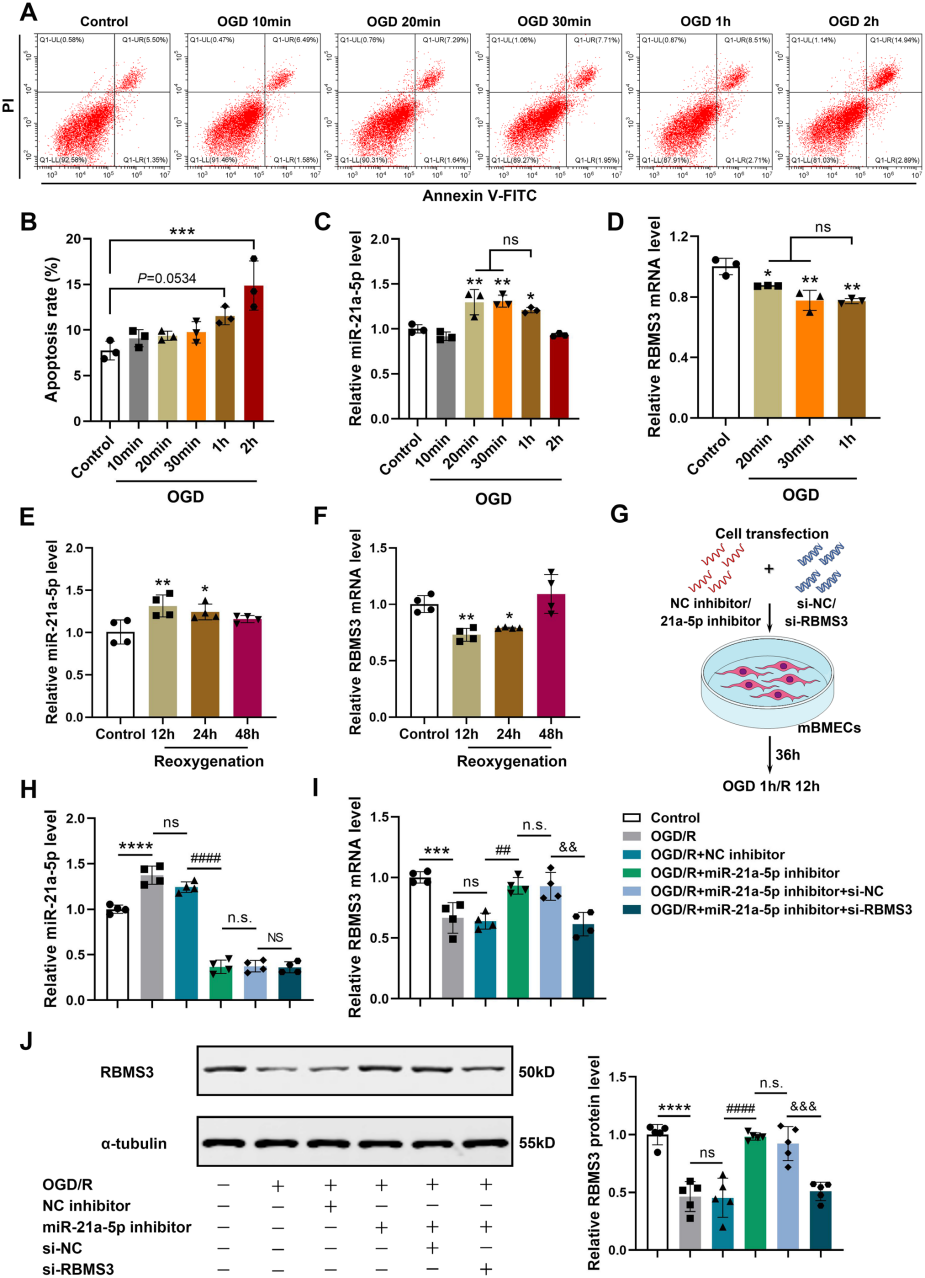
The exposure of cultured mBMECs to OGD for 1 h mimicked TIA‐induced cell damage, and miRNA‐21a‐5p directly targeted RBMS3 expression in vitro. (A) Representative images of apoptotic mBMECs after exposure to OGD for varying durations, as determined by flow cytometry. (B) Quantitative analysis of apoptosis rates (*n* = 3). (C, D) Detection of the mRNA levels of miRNA‐21a‐5p (C) and RBMS3 (D) in mBMECs exposed to OGD for different durations by RT‐qPCR (*n* = 3). (E, F) Detection of the mRNA levels of miRNA‐21a‐5p (E) and RBMS3 (F) in 1‐h OGD‐exposed mBMECs at various time points after reoxygenation by RT‐qPCR (*n* = 4). (G) A schematic diagram showing cell transfection and OGD/R treatment in primary cultured mBMECs. (H) RT‐qPCR analysis of miRNA‐21a‐5p expression in cultured mBMECs after varying treatments (*n* = 4). (I, J) Detection of the mRNA and protein levels of RBMS3 in cultured mBMECs by RT‐qPCR (I) and Western blotting (J) (*n* = 4–5). Data were presented as mean ± SD. *p < 0.05, **p < 0.01, ***p < 0.001, ****p < 0.0001 versus control group; ns, no significance; ^##^ *p* < 0.01, ^####^ *p* < 0.0001 versus OGD/R + NC inhibitor group; n.s., no significance versus OGD/R + miR‐21a‐5p inhibitor group; ^&&^ *p* < 0.01, ^&&&^ *p* < 0.001, NS, no significance versus OGD/R + miR‐21a‐5p inhibitor + si‐NC group by one‐way ANOVA with Tukey's post hoc test.

To develop a suitable TIA model in vitro, primary mBMECs were treated with OGD for varying durations, including 10 min, 20 min, 30 min, 1 h, and 2 h, and cell viability and apoptosis were measured. The findings indicated that the cell viability of mBMECs gradually decreased (Figure S3), and the apoptosis rate increased with the extension of OGD duration (Figure 5A,B). Compared to the control group, the apoptosis rate increased notably in mBMECs treated with OGD for 2 h while exhibiting an increasing trend in the 1‐h OGD group (*p* = 0.0534) (Figure 5B). Moreover, the expression of miRNA‐21a‐5p was markedly elevated in the 1‐h OGD group compared to the control group, accompanied by a decrease in RBMS3 mRNA level. Both changes were consistent with the expression patterns observed in the brains of TIA mice. Whereas, miRNA‐21a‐5p levels exhibited no obvious difference between the 2‐h OGD and control groups (Figure 5C,D). These findings demonstrated that the exposure of primary cultured mBMECs to 1‐h OGD simulated TIA‐induced cellular damages. Then, after 1 h of OGD exposure, mBMECs were reoxygenated for 12, 24, and 48 h, among which the mRNA levels of miRNA‐21a‐5p and RBMS3 changed most significantly at 12 h after reoxygenation (Figure 5E,F). Thus, 12 h of reoxygenation was chosen as the observation time point in subsequent experiments.

As shown in Figure 5G, primary cultured mBMECs were transfected with miRNA‐21a‐5p inhibitor, si‐RBMS3, or corresponding negative controls at 36 h before 1‐h OGD treatment, and the cells were collected after reoxygenation for 12 h. The results of RT‐qPCR and Western blotting revealed that transfection with miRNA‐21a‐5p inhibitor notably decreased miRNA‐21a‐5p levels and increased RBMS3 levels in OGD/R‐exposed mBMECs compared with NC inhibitor transfection. Furthermore, compared to the OGD/R + miRNA‐21a‐5p inhibitor + si‐NC group, miRNA‐21a‐5p levels did not markedly change, but the mRNA and protein levels of RBMS3 were significantly down‐regulated in the OGD/R + miRNA‐21a‐5p inhibitor + si‐RBMS3 group (Figure 5H–J). Collectively, these findings implied that miRNA‐21a‐5p also negatively modulated the expression level of RBMS3 in primary cultured mBMECs, further affirming that RBMS3 was the target of miRNA‐21a‐5p.

### 3.6 MiRNA‐21a‐5p Knockdown Decreased Angiogenesis in the Cellular TIA Model, and RBMS3 Suppression Reversed This Effect

**FIGURE 6:**
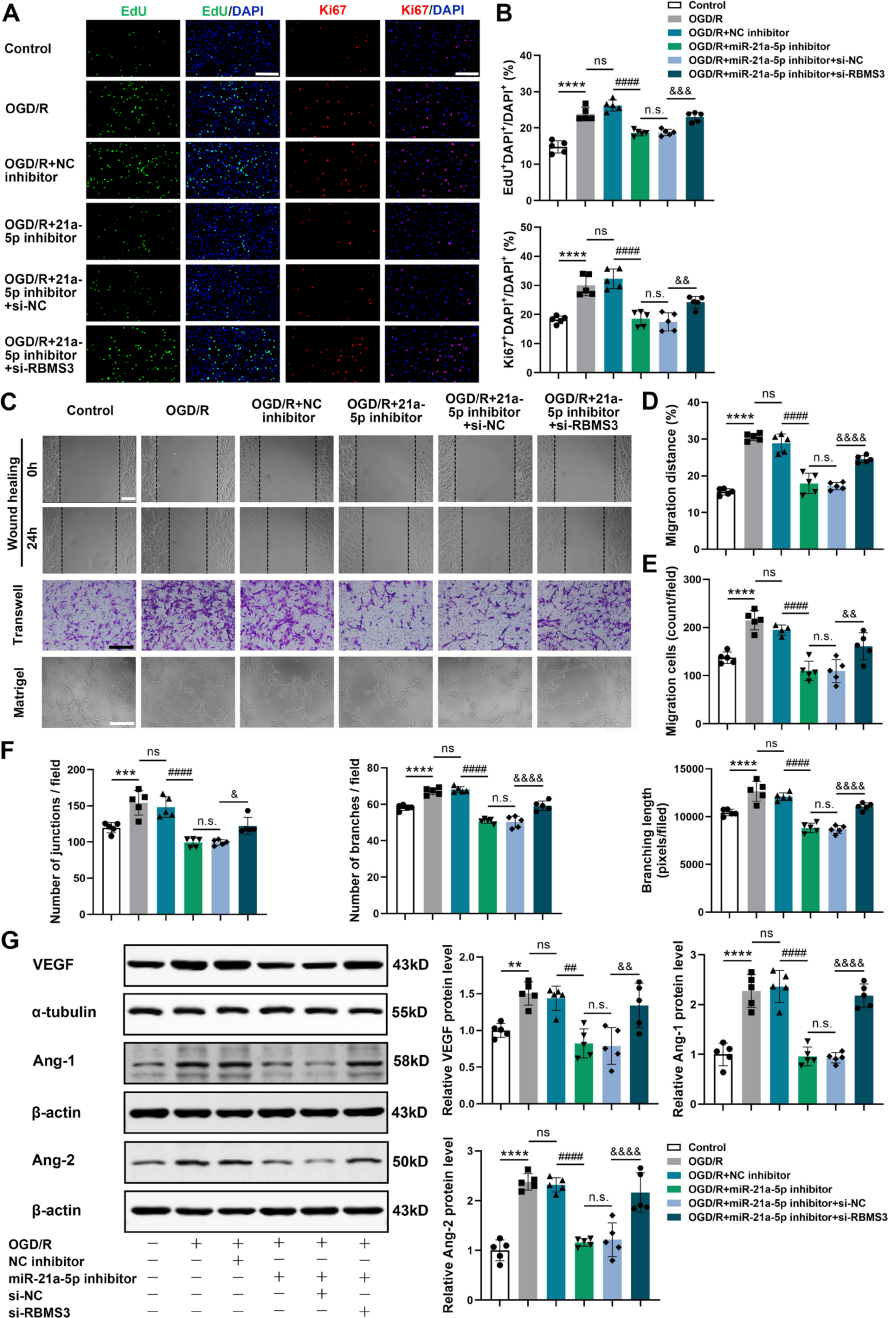
Downregulation of miRNA‐21a‐5p repressed angiogenic activities in the cellular TIA model, and RBMS3 suppression reversed these effects. (A) Representative images of EdU staining and Ki67 immunostaining in primary cultured mBMECs, scale bar = 200 μm. (B) Bar graphs revealing the percentage of EdU‐labeled cells and Ki67‐positive cells in cultured mBMECs (*n* = 5). (C) Representative images of wound healing assay (scale bar = 100 μm), transwell migration assay (scale bar = 100 μm), and Matrigel tube formation assay (scale bar = 200 μm) in cultured mBMECs. (D) Quantification of migration distance detected by wound healing assay (*n* = 5). (E) Quantification of migration cells measured by transwell assay (*n* = 4–5). (F) Quantitative analysis of junction counts, branch numbers, and branching length, as determined by tube formation assay (*n* = 5). (G) Representative Western blotting images of VEGF, Ang‐1, and Ang‐2 in primary cultured mBMECs after different treatments. Bar graphs displaying quantitative data (*n* = 5). Data were presented as mean ± SD. **p < 0.01, ***p < 0.001, ****p < 0.0001 versus control group; ns, no significance versus OGD/R group; ^##^ *p* < 0.01, ^####^ *p* < 0.0001 versus OGD/R + NC inhibitor group; n.s. no significance versus OGD/R + miR‐21a‐5p inhibitor group; ^&^ *p* < 0.05, ^&&^ *p* < 0.01, ^&&&^ *p* < 0.001, ^&&&&^ *p* < 0.0001 versus OGD/R + miR‐21a‐5p inhibitor + si‐NC group by one‐way ANOVA with Tukey's post hoc test.

Since endothelial cell proliferation is deeply involved in angiogenesis, in vitro angiogenesis was measured using EdU proliferation assay and Ki67 immunofluorescent staining. It was found that the proportion of EdU‐labeled and Ki67‐positive proliferative mBMECs was significantly lower in the OGD/R + miRNA‐21a‐5p inhibitor group than that in the OGD/R + NC inhibitor group. Compared to the OGD/R + miRNA‐21a‐5p inhibitor + si‐NC group, the proportion of proliferative cells was markedly increased in the OGD/R + miRNA‐21a‐5p inhibitor + si‐RBMS3 group (Figure 6A,B). We also investigated the effects of miRNA‐21a‐5p and RBMS3 on angiogenesis using wound healing and transwell migration assays with mBMECs. It was found that the migration distance of mBMECs and the number of migrating cells were markedly decreased in the OGD/R + miRNA‐21a‐5p inhibitor group compared with the OGD/R + NC inhibitor group. Compared to the OGD/R + miRNA‐21a‐5p inhibitor + si‐NC group, endothelial cell migration was obviously enhanced in the OGD/R + miRNA‐21a‐5p inhibitor + si‐RBMS3 group (Figure 6C–E). Moreover, Matrigel tube formation assay indicated that in comparison with NC inhibitor transfection, miRNA‐21a‐5p inhibitor transfection remarkably downregulated tube formation in OGD/R‐exposed mBMECs, showing decreased junction counts, branch numbers, and branching length. Compared to the OGD/R + miRNA‐21a‐5p inhibitor + si‐NC group, the tube formation ability of mBMECs was dramatically enhanced in the OGD/R + miRNA‐21a‐5p inhibitor + si‐RBMS3 group, exhibiting elevated junction counts, branch numbers, and branching length (Figure 6C,F).

Furthermore, the expression levels of VEGF, Ang‐1, and Ang‐2 were markedly declined in the OGD/R + miRNA‐21a‐5p inhibitor group compared to the OGD/R + NC inhibitor group. Compared with the OGD/R + miRNA‐21a‐5p inhibitor + si‐NC group, the expression levels of VEGF, Ang‐1, and Ang‐2 were obviously elevated in the OGD/R + miRNA‐21a‐5p inhibitor + si‐RBMS3 group (Figure 6G). Taken together, downregulation of miRNA‐21a‐5p in OGD/R‐exposed mBMECs significantly decreased cell proliferation, migration, and tube formation, and reduced the expression of pro‐angiogenic factors. Moreover, RBMS3 suppression could reverse the inhibitory effects of miRNA‐21a‐5p knockdown on angiogenesis. Therefore, miRNA‐21a‐5p modulated angiogenesis by targeting RBMS3 in the OGD/R‐induced TIA model in vitro.

### 3.7 MiRNA‐21a‐5p Activated the TGFBR1/SMAD2/3 Signaling Pathway by Downregulating RBMS3 in Both Cellular and Animal TIA Models

**FIGURE 7:**
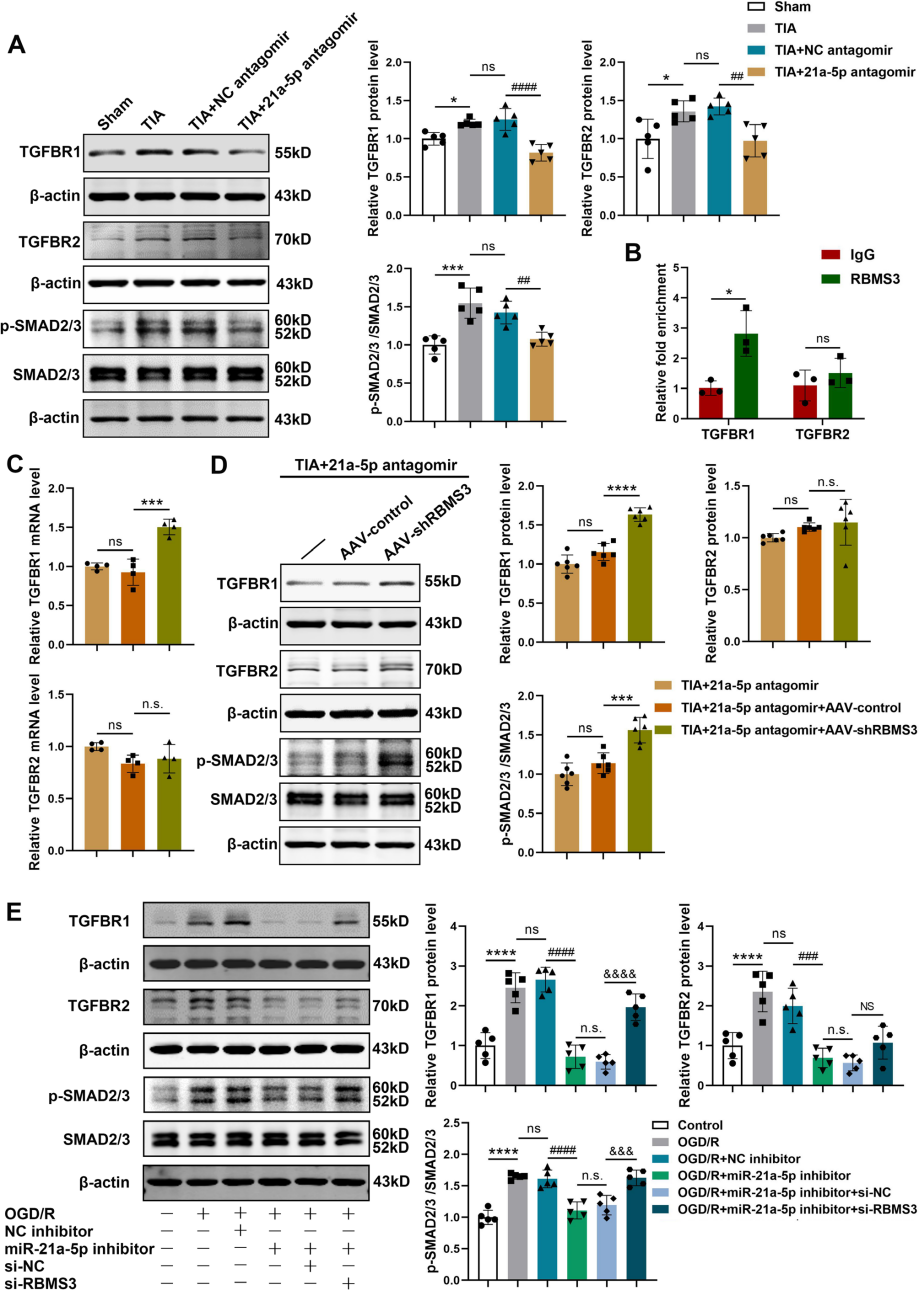
MiRNA‐21a‐5p inhibited RBMS3, thereby activating the TGFBR1/SMAD2/3 signaling pathway in both cellular and animal TIA models. (A) Representative images of Western blotting for TGFBR1, TGFBR2, p‐SMAD2/3 and SMAD2/3 in the brains of the sham, TIA, TIA + NC antagomir and TIA + 21a‐5p antagomir groups. Bar graphs showing data quantification (*n* = 5). (B) Detection of the direct binding between RBMS3 protein and TGFBR1/TGFBR2 mRNA by RIP assay (*n* = 3). (C) Detection of the mRNA levels of TGFBR1 and TGFBR2 in the brain tissues of the TIA + 21a‐5p antagomir, TIA + 21a‐5p antagomir + AAV‐control and TIA + 21a‐5p antagomir + AAV‐shRBMS3 groups by RT‐qPCR (*n* = 4). (D) Representative Western blotting images and protein quantitative analysis of TGFBR1, TGFBR2, p‐SMAD2/3, and SMAD2/3 in the brains of the TIA + 21a‐5p antagomir, TIA + 21a‐5p antagomir + AAV‐control and TIA + 21a‐5p antagomir + AAV‐shRBMS3 groups (*n* = 6). (E) Detection of the protein levels of TGFBR1, TGFBR2, p‐SMAD2/3 and SMAD2/3 in primary cultured mBMECs after different treatments by Western blotting (*n* = 5). Data were presented as mean ± SD. In (A), *p < 0.05, ***p < 0.001 versus sham group; ns, no significance versus TIA group; ^##^ *p* < 0.01, ^####^ *p* < 0.0001 versus TIA + NC antagomir group by one‐way ANOVA with Tukey's post hoc test. In (B), *p < 0.05, ns, no significance versus IgG group by unpaired two‐tailed *t*‐test. In (C and D), ns, no significance versus TIA + 21a‐5p antagomir group; ***p < 0.001, ****p < 0.0001, n.s., no significance versus TIA + 21a‐5p antagomir + AAV‐control group by one‐way ANOVA with Tukey's post hoc test. In (E), ****p < 0.0001 versus control group; ns, no significance versus OGD/R group; ^###^ *p* < 0.001, ^####^ *p* < 0.0001 versus OGD/R + NC inhibitor group; n.s., no significance versus OGD/R + miR‐21a‐5p inhibitor group; ^&&&^ *p* < 0.001, ^&&&&^ *p* < 0.0001, NS, no significance versus OGD/R + miR‐21a‐5p inhibitor + si‐NC group by one‐way ANOVA with Tukey's post hoc test.

To further investigate the downstream pathways of miRNA‐21a‐5p regulating RBMS3‐mediated angiogenesis following TIA, we first detected the effect of miRNA‐21a‐5p on the expression levels of key proteins in the TGF‐β signaling pathway in mouse brains. The results revealed that the levels of TGFBR1 and TGFBR2 proteins and SMAD2/3 phosphorylation were markedly higher in the TIA group than those in the sham group. However, compared to NC antagomir, miRNA‐21a‐5p antagomir dramatically reduced the protein levels of TGFBR1 and TGFBR2 and decreased SMAD2/3 phosphorylation after TIA (Figure 7A). Since RBMS3 encodes an RNA‐binding protein that is responsible for the modulation of gene expression at both the transcriptional and post‐transcriptional levels by binding to mRNAs, the mRNA molecules bound to RBMS3 protein were obtained using RNA binding protein immunoprecipitation (RIP) assay and then detected by RT‐qPCR. We found that TGFBR1 mRNA was markedly enriched in the anti‐RBMS3 group compared to the anti‐IgG group, whereas the enrichment level of TGFBR2 mRNA did not differ significantly between the two groups (Figure 7B). The results suggested that RBMS3 protein could directly bind to TGFBR1 mRNA, but not TGFBR2.

Next, we examined the role of RBMS3 in the TGF‐β signaling pathway in mouse brains after TIA. The RT‐qPCR and Western blotting data unveiled that in TIA mice injected with miRNA‐21a‐5p antagomir, compared with AAV‐control administration, inhibition of RBMS3 expression by AAV‐shRBMS3 markedly upregulated the mRNA and protein levels of TGFBR1 and the phosphorylation level of SMAD2/3, but did not change TGFBR2 expression (Figure 7C,D).

Besides, the specific molecular mechanism by which miRNA‐21a‐5p/RBMS3 regulated angiogenesis was validated in primary cultured mBMECs in vitro. We found that the levels of TGFBR1 and TGFBR2 proteins and SMAD2/3 phosphorylation were remarkably elevated in OGD/R‐exposed mBMECs compared to the control group, but were dramatically decreased in the OGD/R + miRNA‐21a‐5p inhibitor group compared to the OGD/R + NC inhibitor group. Nevertheless, in contrast to the OGD/R + miRNA‐21a‐5p inhibitor + si‐NC group, the levels of TGFBR1 protein and SMAD2/3 phosphorylation markedly increased in the OGD/R + miRNA‐21a‐5p inhibitor + si‐RBMS3 group, with no obvious difference in TGFBR2 expression between the two groups (Figure 7E). Collectively, these results indicated that inhibition of RBMS3 reversed miRNA‐21a‐5p knockdown‐induced decrease in TGFBR1 expression and SMAD2/3 phosphorylation, but did not change TGFBR2 levels. Therefore, miRNA‐21a‐5p could activate the TGFBR1/SMAD2/3 pathway by downregulating RBMS3.

## 4 Discussion

In the present study, we first identified the miRNA with the most significant changes after TIA, namely miRNA‐21a‐5p. Subsequently, we investigated the role of miRNA‐21a‐5p in regulating angiogenesis after TIA and unraveled the underlying molecular mechanisms in rodent and cell models. Our findings included the following: (1) The expression level of miRNA‐21a‐5p was significantly elevated in the brain tissues of TIA mice. Downregulation and upregulation of miRNA‐21a‐5p inhibited and promoted angiogenesis in TIA mice, respectively. (2) 1‐h exposure of cultured mBMECs to OGD simulated the cellular damage induced by TIA. Downregulation of miRNA‐21a‐5p decreased cell proliferation, migration, and tube formation in OGD/R‐exposed mBMECs. (3) RBMS3 was a direct target gene for miRNA‐21a‐5p, which mediated the effect of miRNA‐21a‐5p on angiogenesis after TIA. (4) MiRNA‐21a‐5p activated the downstream TGFBR1/SMAD2/3 signaling pathway by suppressing the expression of the target gene RBMS3 in both cell and animal models of TIA. Our data unveiled that miRNA‐21a‐5p played an essential role in modulating angiogenesis after TIA and served as a promising therapeutic target for TIA.

Currently, most studies on ischemic stroke focus on cerebral infarction, while TIA is rarely discussed. To explore the pathophysiological mechanisms and targeted therapeutic strategies of TIA, we previously developed an optimal TIA model using the modified suture MCAO method considering the three assumptions [5]. The novelty of this model was neither the usage of an especial approach to induce focal cerebral ischemia nor the use of advanced techniques for outcome evaluation, but it was the implementation of three criteria for inducing TIA [3, 4]. The criteria include the following: (i) objective evidence of ischemia/reperfusion, mainly based on CBF measurement to verify sufficient occlusion/reperfusion, (ii) absence of permanent neurological impairment, and (iii) no occurrence of acute cerebral infarction. The second clinical criterion (neurological function score) and the third imaging criterion (MRI examination) are necessary for the TIA model to mimic the episode in clinical patients. These criteria were fulfilled for 8‐min MCAO, proving an efficient TIA model in C57BL/6 mice.

Over the last years, many reports have shown that the expression levels of miRNAs change dramatically after ischemic stroke [21, 22]. In a recent study, Salman et al. performed small RNA sequencing on serum samples of 191 patients with acute cerebral infarction and 61 patients with TIA, and identified 11 miRNAs that could distinguish acute cerebral infarction from TIA [43]. However, the study did not include healthy controls, so it is unclear how miRNA expression changed after TIA compared to the healthy population. In the current study, through small RNA sequencing and RT‐qPCR verification, two differentially expressed miRNAs were identified in the brain tissues of TIA mice relative to sham‐operated mice. Among them, miRNA‐21a‐5p with the most obvious change in expression level was selected for further investigation. At this stage, we did not rule out the involvement of other miRNAs and proteins in the pathogenesis of TIA. These additional mechanisms do not oppose our results and warrant further exploration.

Several angiogenic factors are released after the development of cerebral ischemia. Angiogenesis is a critical protective mechanism promoting the repair of brain injury after TIA. Hence, the therapeutic strategies for TIA should involve the enhancement of angiogenesis in the ischemic area, which can effectively reduce the risk of recurrent ischemic events, improve cognitive and emotional disorders caused by cerebral hypoperfusion [44], and postpone some adverse prognostic events, such as brain atrophy. Herein, we observed that miRNA‐21a‐5p was significantly up‐regulated in TIA mice, suggesting that it might participate in the pathogenesis of TIA. Searching the published literature, miRNA‐21a‐5p was found to be highly expressed in endothelial cells [23, 24, 42]. Moreover, miRNA‐21a‐5p could promote endothelial cell proliferation and migration, and induce angiogenesis in cancer and other non‐neoplastic diseases [26, 27, 29]. However, it remained unknown whether miRNA‐21a‐5p regulates angiogenesis after TIA. Therefore, this study concentrated on the effects and mechanisms of miRNA‐21a‐5p on angiogenesis following TIA. We injected the miRNA‐21a‐5p antagomir or agomir into the contralateral cerebral ventricle of TIA mice and found that angiogenesis was markedly inhibited in the brain after downregulating miRNA‐21a‐5p, whereas miRNA‐21a‐5p overexpression had a completely opposite effect on angiogenesis, suggesting that miRNA‐21a‐5p was responsible for the endogenous regulation of angiogenesis after TIA.

Exploring the endogenous mechanism of angiogenesis is conducive to improving targeted therapy for TIA. Specifically, miRNAs are promising therapeutic targets, which are involved in the pathogenesis of several disorders by modulating the expression levels of multiple genes [45, 46]. Herein, to explore the molecular mechanism through which miRNA‐21a‐5p regulates angiogenesis after TIA, we screened the target genes related to angiogenesis through bioinformatic prediction, transcriptome sequencing, and RT‐qPCR validation. Notably, inhibition of miRNA‐21a‐5p expression only increased the expression levels of RBMS3 and tissue inhibitor of metalloproteinases 3 (TIMP3). In view of the fact that the role of the miRNA‐21a‐5p/TIMP3 axis has previously been identified in the angiogenesis of the central nervous system [47, 48, 49], we selected RBMS3 as a potential target gene of miRNA‐21a‐5p for further investigation. As expected, through dual luciferase reporter gene assay, expression correlation detection, and functional verification, we found that RBMS3 mediated the regulatory effect of miRNA‐21a‐5p on angiogenesis after TIA.

Interestingly, a study reported that RBMS3 regulated chondrogenesis and craniofacial development via the TGF‐β signaling pathway [36], which also plays a key role in angiogenesis [33, 34, 35]. Thus, we investigated whether the TGF‐β pathway was responsible for the modulation of the miRNA‐21a‐5p/RBMS3 axis on angiogenesis after TIA. RIP analysis revealed that RBMS3 protein was directly bound to TGFBR1 mRNA, but not to TGFBR2 mRNA. Consistently, we observed that repression of the target gene RBMS3 only reversed the decrease in TGFBR1 expression and SMAD2/3 phosphorylation after miRNA‐21a‐5p knockdown, but did not influence TGFBR2 level. This finding indicated that miRNA‐21a‐5p upregulated TGFBR1 expression by suppressing the target gene RBMS3, thereby upregulating the phosphorylation level of SMAD2/3. However, another study showed that SMAD2 mRNA could be stabilized by directly binding to RBMS3 protein during chondrogenesis, and RBMS3 knockdown in embryos reduced SMAD2 activation, thereby resulting in severe craniofacial defects [36]. This might be ascribed to the presence of more than one binding site for RBMS3 protein on SMAD2 mRNA. The final 150 bp region of SMAD2 3′UTR was determined to contain RBMS3‐regulated stabilization sites, whereas the first 200 bp region was found to mediate the destabilization or even degradation of the SMAD2 transcript [36], suggesting two functional domains on the SMAD2 mRNA that mediated positive and negative regulation of RBMS3. In other words, RBMS3 can promote or inhibit gene expression, relying largely on the binding site of RBMS3 protein for the target mRNA. In the present study, the RBMS3 protein might be primarily bound to the negative functional domain of TGFBR1 mRNA, thereby reducing its stability and leading to its degradation. It is worthy of further investigation whether the SMAD2 level is directly regulated by RBMS3 after TIA.

The neurovascular unit is composed of neurons, glial cells, and blood vessels, which are structurally and functionally related to maintain the homeostasis of the brain. As a crucial member of the neurovascular unit, brain microvascular endothelial cells (BMECs) interact closely with other cells, thereby providing a stable internal microenvironment for nerve function. Cerebral ischemia immediately damages BMECs, resulting in blood–brain barrier injury and vascular dysfunction. Moreover, BMECs generate various signaling molecules affecting brain repair [50, 51]. In view of the important role of BMECs in cerebral ischemic injury and repair, we evaluated the outcomes after exposure to OGD for varying durations to establish a suitable TIA model in vitro and investigated the direct effect of miRNA‐21a‐5p on angiogenesis in cultured BMECs. Our findings showed that the apoptosis rate exhibited an increasing trend in mBMECs exposed to OGD for 1 h. Following the three established criteria for the in vivo TIA model [3, 4], we selected 1‐h OGD exposure to simulate the cerebral ischemia environment following TIA in vitro. Instead, OGD of 2 h was a critical threshold that could induce distinct apoptosis of mBMECs. Additionally, the miRNA‐21a‐5p level markedly increased in primary cultured mBMECs after 1‐h OGD. Similar changes in miRNA‐21a‐5p expression were observed in TIA mice, indicating that the cellular model was suitable for in vitro studies of TIA. It is worth noting that the expression level of miRNA‐21a‐5p is markedly different in disparate cells after OGD/R. In primary cultured neurons, the expression of miRNA‐21a‐5p was significantly reduced after 3 h of OGD [52]. Similarly, the level of miRNA‐21a‐5p was down‐regulated in BV2 cells treated with OGD for 12 h [53]. In contrast, the expression of miRNA‐21a‐5p increased in a time‐dependent manner at different time points in OGD‐exposed neural stem cells [54]. Besides, the levels of miRNA‐21a‐5p were dramatically elevated in primary cultured neurons and astrocytes after exposure to OGD for 1 h [55]. The mechanism underlying this variation may be the temporal difference in miRNA expression, which is dynamically regulated by several factors, including tissue characteristics, disease stages, and stimulus duration.

In conclusion, our findings indicated that miRNA‐21a‐5p targeted RBMS3 and then modulated the downstream TGFBR1/SMAD2/3 pathway to affect the angiogenic activities of mBMECs and regulate angiogenesis after TIA. As far as we know, this is the inaugural study to discover the endogenous regulatory role of miRNA‐21a‐5p in angiogenesis after TIA. These data highlighted the potential application of miRNA‐21a‐5p as a therapeutic target for TIA and provided innovative evidence for pro‐angiogenic therapy in patients with TIA.

## Author Contributions

Jiahui Wang: investigation, methodology, data curation, writing – original draft. Yanyan Li: data curation, methodology. Jingyi Wang and Shiling Chen: visualization, formal analysis. Luwei Nie, Xuan Wu, and Jiarui Li: software, validation. Ping Zhang and Zhouping Tang: conceptualization, supervision, funding acquisition, writing – review and editing. All authors read and approved the final manuscript.

## Ethics Statement

All animal experiments were approved by the Experimental Animal Ethics Committee of Tongji Hospital, Tongji Medical College, Huazhong University of Science and Technology (TJ‐IRB20211270).

## Conflicts of Interest

The authors declare no conflicts of interest.

## Supporting information


**Data S1:** cns70573‐sup‐0001‐DataS1.pdf.
**Figure S1:** Construction and validation of the mouse model of TIA. (A) Representative laser speckle images revealing CBF before surgery (baseline), after occlusion (MCAO), and after reperfusion. (B) Representative laser Doppler flow images of the ipsilateral cerebral hemisphere. The results showed that the CBF values of right hemisphere immediately dropped below 20% of the baseline levels after initiating MCAO, and the blood perfusion significantly recovered after suture withdrawal (Criterion 1). (C) Representative coronal brain MRI images (T2WI and DWI sequences) at 24 h following the onset of reperfusion. The results revealed that neither obvious neurological deficits (Criterion 2) nor MRI detectable cerebral infarct lesions (Criterion 3) were detected at 24 h of reperfusion, indicating that the TIA model was successfully established.
**Figure S2:** Differentially expressed miRNAs in the TIA and sham‐operated groups identified by small RNA sequencing. (A) The volcano plot of differentially expressed miRNAs in TIA mice and sham‐operated mice. (B) The expression levels of miRNAs in TIA mice and sham‐operated mice quantified by small RNA sequencing (|log_2_FoldChange| > 1 and *Q*‐value < 0.05).
**Figure S3:** Cell viability gradually decreased with the extension of OGD duration in primary cultured mBMECs. (A) Representative images of bright field and vWF immunofluorescent staining in primary cultured mBMECs, scale bar = 50 μm. (B) Detection of cell viability in primary cultured mBMECs after exposure to OGD for different durations by CCK‐8 assay (*n* = 4–5). Data were presented as mean ± SD. ***p < 0.001, ****p < 0.0001 versus control group by one‐way ANOVA with Tukey's post‐hoc test.
**Table S1:** List of primer sequence used for the qPCR reaction. *The reverse primers of miRNAs were provided by the miRcute miRNA qPCR Kit (TIANGEN, China), in which the sequences were not disclosed. qPCR, quantitative real‐time PCR.
**Table S2:** List of primary antibodies applied in the present study. IF, immunofluorescent staining; WB, Western blot; RIP, RNA binding protein immunoprecipitation.
**Table S3:** The mRNA levels of 20 candidate target genes related to angiogenesis in TIA mice versus sham‐operated mice quantified by transcriptome sequencing.

**Data S2:** cns70573‐sup‐0002‐DataS2.pdf.

## Data Availability

The data that support the findings of this study are available from the corresponding author upon reasonable request.
