# Supplementary material for: MicroRNA‐21a‐5p Promotes Cerebral Angiogenesis in Transient Ischemic Attack by Targeting RBMS3 and Subsequently Modulating the TGFBR1/SMAD2/3 Pathway
Source: CNS Neurosci Ther. 2025 Aug 18;31(8):e70573. doi: 10.1111/cns.70573 (PMC12358734; doi:10.1111/cns.70573)
Supplement: Supplementary file 1 — Data S1: cns70573‐sup‐0001‐DataS1.pdf. Figure S1: Construction and validation of the mouse model of TIA. (A) Representative laser speckle images revealing CBF before surgery (baseline), after occlusion (MCAO), and after reperfusion. (B) Representative laser Doppler flow images of the ipsilateral cerebral hemisphere. The results showed that the CBF values of right hemisphere immediately dropped below 20% of the baseline levels after initiating MCAO, and the blood perfusion significantly recovered after suture withdrawal (Criterion 1). (C) Representative coronal brain MRI images (T2WI and DWI sequences) at 24 h following the onset of reperfusion. The results revealed that neither obvious neurological deficits (Criterion 2) nor MRI detectable cerebral infarct lesions (Criterion 3) were detected at 24 h of reperfusion, indicating that the TIA model was successfully established. Figure S2: Differentially expressed miRNAs in the TIA and sham‐operated groups identified by small RNA sequencing. (A) The volcano plot of differentially expressed miRNAs in TIA mice and sham‐operated mice. (B) The expression levels of miRNAs in TIA mice and sham‐operated mice quantified by small RNA sequencing (|log2FoldChange| > 1 and Q‐value < 0.05). Figure S3: Cell viability gradually decreased with the extension of OGD duration in primary cultured mBMECs. (A) Representative images of bright field and vWF immunofluorescent staining in primary cultured mBMECs, scale bar = 50 μm. (B) Detection of cell viability in primary cultured mBMECs after exposure to OGD for different durations by CCK‐8 assay (n = 4–5). Data were presented as mean ± SD. ***p < 0.001, ****p < 0.0001 versus control group by one‐way ANOVA with Tukey's post‐hoc test. Table S1: List of primer sequence used for the qPCR reaction. *The reverse primers of miRNAs were provided by the miRcute miRNA qPCR Kit (TIANGEN, China), in which the sequences were not disclosed. qPCR, quantitative real‐time PCR. Table S2: List of primary antibo [file CNS-31-e70573-s002.pdf]

Supplementary Material for

MicroRNA-21a-5p promotes cerebral angiogenesis in transient ischemic attack by targeting RBMS3 and subsequently modulating the TGFBR1/SMAD2/3 pathway

Jiahui Wang[1, 2], Yanyan Li[1], Jingyi Wang[1], Shiling Chen[1], Luwei Nie[1], Xuan Wu[1], Jiarui Li[1], Ping Zhang[1, *], Zhouping Tang[1, *]

* Correspondence:

Ping Zhang (ppkitty0609@163.com), Zhouping Tang (ddjtzp@163.com)

Department of Neurology, Tongji Hospital, Tongji Medical College, Huazhong University of Science and Technology, No.1095 Jiefang Avenue, Qiaokou District, Wuhan 430030, China

This file includes:

Supplementary Materials and Methods

Figures S1-S3

Tables S1-S3

Supplementary Materials and Methods

Operation of middle cerebral artery occlusion (MCAO)

Briefly, mice were anesthetized with isoflurane (2.5% for induction, 1.5% for maintenance) diluted in oxygen (97.5%). A midline neck incision was made to expose the right common carotid artery, external carotid artery, and internal carotid artery. A monofilament with a silicone rubber-coated tip (Jialing Biotech Co., Ltd, Guangzhou, China) was gently inserted through the external carotid artery into the internal carotid artery and further to the origin of the middle cerebral artery (MCA). After 8 min of MCA occlusion, reperfusion was achieved by retracting the suture. In sham-operated mice, similar operations were performed without inserting the suture. During the procedure, the rectal temperature of each mouse was kept at 37.0 ± 0.5 °C using a heating pad.

Cerebral blood flow (CBF) measurement by laser speckle contrast imaging

Mice were anesthetized with isoflurane and their heads were secured in a prone position on a stereotaxic frame. After making a midline incision on the scalp to expose the skull, the mouse was positioned under a microscope connected to the laser speckle imaging system (Xunwei Opto-Technology Co., Ltd, Wuhan, China). The focal length was adjusted until the blood vessels on the skull surface were clearly observed. Subsequently, the skull surface was illuminated using a 550-nm laser diode in a diffuse and uniform manner. CBF was measured by speckle contrast. Two-dimensional blood flow maps were captured using the system's own image software at 5 min before MCAO, 5 min after the onset of MCAO, and 10 min after

reperfusion. For each time point, images were collected for 1 min, including sixty consecutive images. For image data analysis, an identical and elliptical region of interest (ROI) was created within the ipsilateral MCA blood supply area to measure CBF in each image. Then, CBF was normalized to the pre-operative level to obtain a relative CBF value.

CBF monitoring by laser Doppler flowmeter

Under isoflurane anesthesia, the skull of mice was exposed through a midline incision of the scalp. Then, a fiber-optic probe was secured on the skull surface of the ipsilateral MCA blood supply region. CBF was assessed using the laser Doppler flowmeter (Moor Instruments, UK) at 5 min before MCAO, 5 min after the onset of MCAO, and 10 min after reperfusion. For each time point, CBF was monitored for 1 min, and then CBF was normalized to the pre-operative level to obtain a relative CBF value.

Neurological evaluation

The neurological status of each mouse was evaluated using the Zea-Longa 5-point score scale at 24 h after the onset of reperfusion. The scoring criteria were defined as follows: score 0: no neurological deficit; score 1: failure to fully extend the contralateral forepaw; score 2: walking gait circling to the contralateral side; score 3: falling to the contralateral side; score 4: no autonomous walking with depressed consciousness.

7.0 T magnetic resonance imaging (MRI) examination

Following neurological assessment, a coronal brain MRI scanning, including diffusion weighted imaging (DWI) and T2WI sequences, was conducted on anesthetized mice using a 7.0 T MRI scanner (Bruker Biospect, Germany). A linear birdcage radio frequency coil with 19-mm inner diameter was employed. DWI was obtained by an echo planar imaging sequence (layer thickness = 0.8mm, spatial resolution = 0.156mm), and T2WI was conducted using a T2-weighted rapid acquisition with a relaxation enhancement sequence (layer thickness = 0.8mm, spatial resolution = 0.078mm). The images were visually assessed to identify potential signal-enhanced areas.

Brain tissue preparation

The mice were deeply anesthetized and then transcardially perfused with normal saline. For reverse transcription quantitative PCR (RT-qPCR) and Western blotting, the brains were removed and then cut into 0.5 mm-thick sections using a rodent brain mold. After the surrounding brain tissues were carefully isolated, the brain tissues in the ipsilateral MCA blood supply area (the gray zone in Figure 1D) were collected and rapidly frozen in liquid nitrogen. For immunofluorescent staining, the mice were then perfused with 4% paraformaldehyde (PFA). The brain tissues were removed and post-fixed in 4% PFA overnight at 4 °C. After dehydration in a gradient of 20% and 30% sucrose, coronal 15 μm-thick sections of the brain were prepared using a thermostatic freezing microtome (CM 1950, Leica, Germany), and then gently mounted onto microslides. The brain sections were stored at -80 °C before staining.

Transcriptome profiling and bioinformatic analysis

On the 3rd day after the surgery, the brain tissues of transient ischemic attack (TIA) mice and sham-operated mice were collected and placed in liquid nitrogen. Subsequently, the specimens were delivered to Novogene Corporation (Beijing, China) for RNA sequencing using the Illumina HiSeqTM2500 platform. All specimens were qualified for further analyses. Differentially expressed mRNAs or miRNAs were identified using DESeq2 software based on the negative binomial distribution. The volcano map and clustering heatmap were plotted using NovoMagic online tools (https://magic.novogene.com).

RT-qPCR

Total RNA in the brain tissues or cultured cells was isolated using the miRcute miRNA Isolation Kit (TIANGEN, China) according to the manufacturer's instructions. The reverse transcription of miRNA was performed by the miRNA First-Strand cDNA Kit (TIANGEN, China) using poly (A) tailing reaction, while the reverse transcription of mRNA was conducted using the universal cDNA Synthesis Kit (TIANGEN, China). The expression of miRNAs and mRNAs were detected by the miRcute miRNA qPCR Kit and SuperReal PreMix Kit (SYBR Green) (TIANGEN, China), respectively, using the ABI StepOnePlus Real-Time PCR system (Applied Biosystems, USA). U6 and β-actin were served as internal references for miRNA and mRNA quantification, respectively. The relative expression of miRNA or mRNA was determined using the $2^{-\Delta\Delta CT}$ method. The sequences of primers utilized for quantitative real-time PCR (qPCR) were presented in Table S1.

Western blotting

Total proteins of the brain tissues or cultured cells were obtained using RIPA lysis buffer (Beyotime, China) containing protease inhibitor, phosphatase inhibitor (Cell Signaling Technology, USA), and phenylmethanesulfonyl fluoride. Protein concentration was determined using the BCA protein assay kit (Beyotime, China). Equal amounts of protein were separated through 10% electrophoresis gels (SDS-PAGE), and then transferred to nitrocellulose (NC) membranes (PALL, USA). Subsequently, the membranes were blocked with 5% skimmed milk or 5% bovine serum albumin (BSA) for 1 h at room temperature, and then exposed to primary antibodies (Table S2) overnight at 4 °C. After incubation with secondary antibodies (Cell Signaling Technology, USA) for 1 h at room temperature, the protein bands were observed using the Odyssey infrared imaging system (LI-COR Biosciences, USA) or chemiluminescence imaging system (Bio-Rad, USA). The intensity of each band was analyzed using Image J software. The results of Western blotting showed that the bands were of good quality, with clean background, no nonspecific bands, and consistent internal references. In addition, the location of each band was specific and the molecular weight was consistent.

Immunofluorescent staining

Brain slices were permeabilized with 0.25 % Triton X-100 in PBS and then blocked with 10% BSA for 1 h at room temperature. Next, the slices were incubated with primary antibodies (Table S2) overnight at 4 °C, followed by incubation with corresponding secondary antibodies (Jackson, USA) for 1 h at room temperature and counterstaining with DAPI (Servicebio, China).

For negative controls, primary antibodies were replaced with PBS. The stained sections were observed under a fluorescent microscope or a laser scanning confocal microscope (Olympus, Japan). Three brain sections from each mouse were used for analysis. The number of positive labeled cells was counted using ImageJ software.

Fluorescein isothiocyanate (FITC) perfusion imaging

FITC, a derivative of fluorescein, specifically binds to vascular endothelial cells under alkaline conditions and is commonly used to mark cerebral microvessels with blood perfusion (functional blood vessels). Briefly, following deep anesthesia, the mice were transcardially perfused with 100 ml of 10 mM glucose in PBS. After perfusion with 50 ml of 0.1 mg/ml FITC in PBS supplemented with 10 mM glucose, mice were perfused with 10 ml of 5 U/ml heparin, and then with 100 ml of 4% PFA (pH 8.0). The brain tissues were harvested, followed by post-fixing in 4% PFA (pH 8.0) at 4 °C for 24 h and dehydration in 30% sucrose (pH 8.0). Next, the brain tissues were cut into coronal slices with a thickness of 20 μm and mounted onto microslides. The slices were examined using a fluorescent microscope (Olympus, Japan), and the number of microvessels was calculated using ImageJ software.

Dual luciferase reporter gene assay

Mouse brain microvascular endothelial cell line BEND3 was supplied by the Cell Bank of the Chinese Academy of Sciences (Shanghai, China). To confirm the bond between miRNA-21a-5p and 3'UTR of RBMS3 mRNA, cultured BEND3 cells were transfected with a luciferase

vector containing the wild-type (WT) or mutant-type (MUT) 3'UTR sites of RBMS3 (GENERAL BIOL, China), together with miRNA-21a-5p mimic or NC mimic (RiboBio, China) for 24 h. The luciferase activity was evaluated using the Dual-Luciferase® Reporter Assay Systema (Promega, USA) according to the manufacturer's protocol. The outcomes were presented as the ratio of firefly luciferase activity to renilla luciferase activity.

Primary mouse brain microvascular endothelial cells (mBMECs) culture

Briefly, the brains of mice were acquired and the gray matters were cut into small pieces. The tissues were then resuspended with 25% BSA and centrifugated. Thereafter, the sediment in the lowest layer was transferred and digested with 0.1% type II collagenase at 37 °C for 30 min. The microvascular fragments and single endothelial cells were obtained in DMEM/F12 medium containing 20% fetal bovine serum (FBS). Primary mBMECs were cultured in a cell culture incubator with 5% CO_2 at 37 °C (Thermo Fisher Scientific, USA), and the medium was renewed with endothelial culture medium (Sciencell, USA) containing 5% FBS, 1% endothelial growth supplement, and 1% penicillin/streptomycin solution every 2-3 days. Cell passages were performed when the cell density reached 90% confluency. Briefly, the primary mBMECs were rinsed with PBS and then digested using 0.25% trypsin for 2 min at 37 °C. The digested cells were centrifuged at 1000 rpm for 5 min and then subcultured at 1:2. The purity of primary mBMECs was assessed by immunofluorescent staining using an antibody against vWF (Proteintech, China) as the biomarker (Figure S3A). The mBMECs (3-4 passages, > 95% purity) were grown to 90–95% confluency before use.

Apoptosis analysis by flow cytometry

The apoptotic rate of mBMECs was evaluated by flow cytometry using the Annexin V-FITC/PI Apoptosis Detection Kit (BD Biosciences, USA). Briefly, single-cell suspensions were dark incubated with annexin V-FITC for 15 min at room temperature. Subsequently, they were mixed gently with propidium iodide (PI), and analyzed using a flow cytometer (Beckman Coulter, USA).

Cell viability assay

Primary mBMECs were seeded into 96-well plates with 1×10^4 cells per well. After oxygen glucose deprivation/reoxygenation (OGD/R) treatment, the cell viability was determined using the Cell Counting Kit-8 (CCK-8) (Dojindo, Japan). The 96-well plates were incubated at 37 °C for 2 h, and then the absorbance at 450 nm was recorded.

5-Ethynyl-2'-deoxyuridine (EdU) cell proliferation assay

The proliferation of cells was evaluated by EdU staining using the Cell-Light™ EdU Apollo® 488 In Vitro Kit (RiboBio, China). mBMECs were incubated with EdU at a final concentration of 50 μM for 2 h, and then fixed with 4% PFA for 30 min. After washing with 2 mg/ml glycine and permeabilizing by 0.5 % Triton X-100, cells were treated with Apollo staining reaction solution at 37 °C for 30 min, followed by counterstaining with DAPI. Images of the stained cells were acquired using a fluorescent microscope, and the number of EdU-positive cells was measured using Image J software.

Wound healing assay

Primary mBMECs were seeded into 6-well plates at a density of 5×10^5 cells/well. After transfection and OGD/R treatment, wound healing assay was performed to evaluate the horizontal migration of mBMECs. Using a 200-μl pipette tip, a straight line was drawn through the cell monolayers with uniform force. Subsequently, the cells were gently rinsed 3 times with PBS and incubated in a serum-free medium. The scratch gaps were examined and images were acquired using an inverted microscope (Olympus, Japan) at 0 h and 24 h. The average width of scratches was measured using ImageJ software, and the relative distance of cell migration was determined as follows: Migration distance (%) = (Width $_{0h}$ – Width $_{24h}$) / Width $_{0h}$。

Transwell migration assay

The vertical migration of primary mBMECs was assesed by transwell assay. After transfection and OGD/R treatment, 6×10^4 cells suspended in 200 μl serum-free medium were seeded into the upper chamber (Corning, USA). Then, 800 μl of medium containing 10% FBS was added to the lower chamber. After 12 h of culturing, the non-migrated cells on the top surface of the membrane were gently removed with a cotton swab, and the underside of the membrane was fixed with 4% PFA for 20 min and stained with 0.1% crystal violet for 10 min. Cells on the lower surface of the membrane were photographed under an inverted microscope, and quantified using Image J software.

Matrigel tube formation assay

Matrigel (Corning, USA) was used to evaluate the tube formation ability of primary mBMECs. Prechilled 96-well plates were coated with Matrigel (50 μl/well), followed by incubation at 37 °C until solidification. mBMECs (4×10^4 cells/well) were resuspended in 100 μl of complete medium and then seeded on Matrigel-coated 96-well plates. Subsequently, cells were cultured at 37 °C for 4 h, and then the formation of tubular structures was photographed using an inverted microscope. The “Angiogenesis Analyzer” tool of Image J software was applied to measure the number of branch points and total tube length.

RNA binding protein immunoprecipitation (RIP) assay

RIP assay was carried out to analyze the interaction between RBMS3 and TGFBR1 using the EZ-Magna RIP™ RIP Kit (Millipore, USA) according to the manufacturer’s instructions. Briefly, the brain tissues were homogenized in complete RIP lysis buffer. 10 μl of the tissue lysate was served as “10% Input” (used as an input control), and the remaining lysate was incubated with magnetic beads conjugated with anti-RBMS3 (Affinity Biosciences LTD, China) or anti-IgG (used as a control) overnight at 4°C. Co-precipitated RNAs were isolated, purified, and then detected using RT-qPCR.

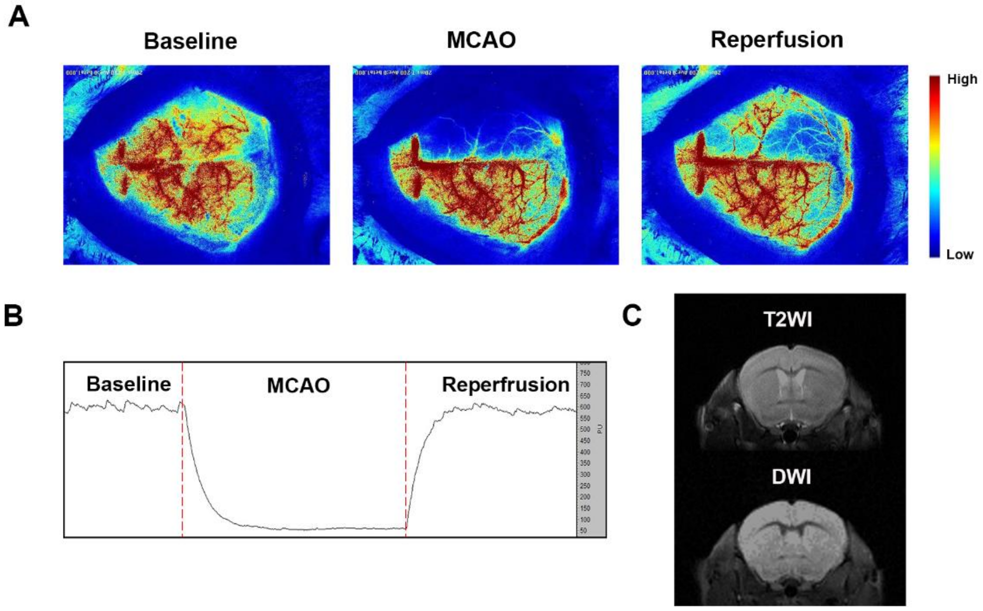

Figure S1. Construction and validation of the mouse model of TIA. **(A)** Representative laser speckle images revealing CBF before surgery (baseline), after occlusion (MCAO), and after reperfusion. **(B)** Representative laser Doppler flow images of the ipsilateral cerebral hemisphere. The results showed that the CBF values of right hemisphere immediately dropped below 20% of the baseline levels after initiating MCAO, and the blood perfusion significantly recovered after suture withdrawal (Criterion 1). **(C)** Representative coronal brain MRI images (T2WI and DWI sequences) at 24 h following the onset of reperfusion. The results revealed that neither obvious neurological deficits (Criterion 2) nor MRI detectable cerebral infarct lesions (Criterion 3) were detected at 24 h of reperfusion, indicating that the TIA model was successfully established.

A

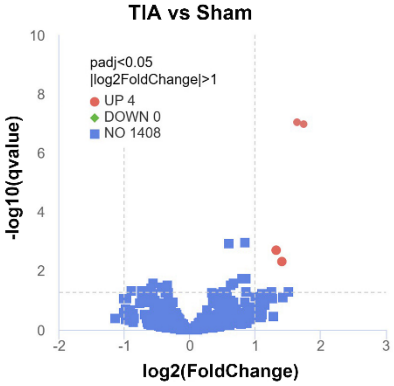

B

TIA vs Sham (|log2FoldChange|>1 and Q-value<0.05)

miRNA	log2FoldChange	Q-value	Regulation
mmu-miR-21a-5p	1.753597841	9.49E-08	Up
mmu-miR-155-5p	1.650960297	6.80E-08	Up
novel_92	1.409251802	0.0065	Up
mmu-miR-10a-5p	1.335090308	0.0027	Up

Figure S2. Differentially expressed miRNAs in the TIA and sham-operated groups identified by small RNA sequencing. **(A)** The volcano plot of differentially expressed miRNAs in TIA mice and sham-operated mice. **(B)** The expression levels of miRNAs in TIA mice and sham-operated mice quantified by small RNA sequencing (|log2FoldChange| > 1 and Q-value < 0.05).

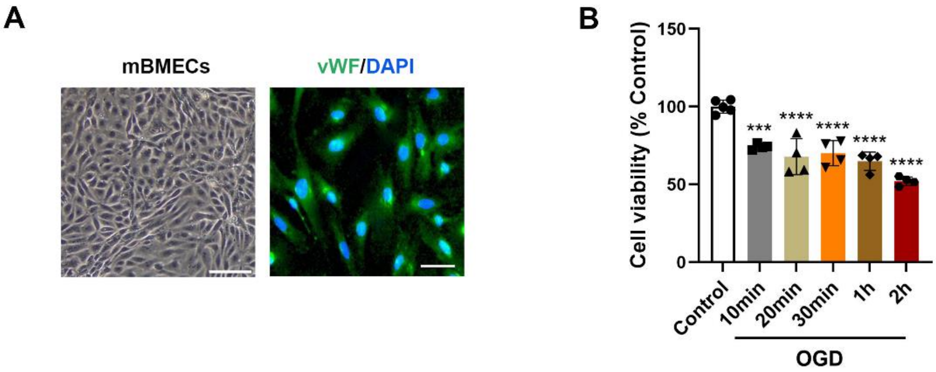

Figure S3. Cell viability gradually decreased with the extension of OGD duration in primary cultured mBMECs. **(A)** Representative images of bright field and vWF immunofluorescent staining in primary cultured mBMECs, scale bar = 50 μm. **(B)** Detection of cell viability in primary cultured mBMECs after exposure to OGD for different durations by CCK-8 assay (n = 4-5). Data were presented as mean ± SD. ***P < 0.001, ****P < 0.0001 versus control group by one-way ANOVA with Tukey's post-hoc test.

Table S1. List of primer sequence used for the qPCR reaction.

Gene	Forward primer (5'-3')	Reverse primer (5'-3')
mmu-miR-21a-5p	CGGCTAGCTTATCAGACTGATGTTGA	– *
mmu-miR-155-5p	CGCTTAATGCTAATTGTGATAGGGGT	– *
mmu-miR-10a-5p	CGTACCCTGTAGATCCGAATTTGTG	– *
U6	TGGAACGATACAGAGAAGATTAGCA	– *
Timp3	GCGTGTATGAAGGCAAGATGTAC	AGGCGTAGTGTTTGGACTGATAG
Epha4	TGGTCCAGGCTAAAGAAGTTACA	CAGAGGGTTCAGGCCTTTGATAT
Hipk3	AGTTGTGCCAGAACAGGAGTAAT	GAGTCGGCAATGATGATGGTTTG
Peli1	ATGGCGAACTCATTGTCTTAGGA	ATGCTGGTCCTTGTTGCTTATTG
Rbms3	GACCAGGACCTCATCAAGCTATG	ATTCGTAGGGTCTTGCTCTTGTT
Tgfbr1	AACCGCACTGTCATTCACCA	ACCGACCTTTGCCAATGCTT
Tgfbr2	CATCAACCACAACACGGAGC	CCACGGTCTCAAACTGCTCT
β-actin	TGGAATCCTGTGGCATCCATGA	AATGCCTGGGTACATGGTGGTA

*The reverse primers of miRNAs were provided by the miRcute miRNA qPCR Kit (TIANGEN, China), in which the sequences were not disclosed.

qPCR: quantitative real-time PCR

Table S2. List of primary antibodies applied in the present study.

Antibody	Host species	Manufacturer	Catalog #	Application/ Working dilution
VEGFA	Rabbit	Abcam, USA	ab214424	WB/ 1:1500
Ang-1	Rabbit	Proteintech, China	23302-1-AP	WB/ 1:1000
Ang-2	Rabbit	Abcam, USA	ab155106	WB/ 1:1000
RBMS3	Rabbit	ABclonal, China	A17142	WB/ 1:1000
TGFBR1	Rabbit	Abcam, USA	ab235578	WB/ 1:1500
TGFBR2	Mouse	Proteintech, China	66636-1-Ig	WB/ 1:6000
SMAD2/3	Rabbit	Cell Signaling Technology, USA	8685	WB/ 1:1000
p-SMAD2/3	Rabbit	Cell Signaling Technology, USA	8828	WB/ 1:1000
GAPDH	Mouse	Proteintech, China	60004-1-Ig	WB/ 1:10000
β-actin	Mouse	Cell Signaling Technology, USA	3700	WB/ 1:1000
β-actin	Rabbit	Cell Signaling Technology, USA	4970	WB/ 1:1000
α-tubulin	Mouse	Cell Signaling Technology, USA	3873	WB/ 1:1000
CD31	Goat	R&D Systems, USA	AF3628	IF/ 1:50
Ki67	Rabbit	Abcam, USA	ab15580	IF/ 1:200
vWF	Rabbit	Proteintech, China	27186-1-AP	IF/ 1:100
RBMS3	Rabbit	Affinity Biosciences, China	DF8599	RIP/ −

WB: Western blot; IF: immunofluorescent staining; RIP: RNA binding protein immunoprecipitation

Table S3. The mRNA levels of 20 candidate target genes related to angiogenesis in TIA mice versus sham-operated mice quantified by transcriptome sequencing.

Gene symbol	Gene description	log2FoldChange	P-value	Q-value
Epha4	Eph receptor A4	-0.51795	3.23E-05	0.000764
Rbms3	RNA binding motif single stranded interacting protein 3	-0.40064	0.005506	0.028648
Peli1	Pellino 1	-0.34869	0.000292	0.003574
Hipk3	Homeodomain interacting protein kinase 3	-0.26074	0.003568	0.021254
Timp3	Tissue inhibitor of metalloproteinase 3	-0.22425	0.02501	0.085435
Spry2	Sprouty RTK signaling antagonist 2	-0.21863	0.040594	0.121463
Reck	Reversion-inducing-cysteine-rich protein with kazal motifs	-0.26685	0.052932	0.146163
Nfib	Nuclear factor I/B	-0.13883	0.233068	0.412708
Sox5	SRY (sex determining region Y)-box 5	-0.12863	0.16769	0.330143
Elf2	E74-like factor 2	-0.06734	0.48457	0.663051
Hnrnpk	Heterogeneous nuclear ribonucleoprotein K	-0.0507	0.511585	0.684665
Pdcd4	Programmed cell death 4	-0.03435	0.677641	0.809327
Btg2	B cell translocation gene anti-proliferation factor 2	-0.02952	0.903879	0.950199

Smad7	SMAD family member 7	0.093326	0.325127	0.515786
Jag1	Jagged 1	0.113535	0.525464	0.695757
Yap1	Yes-associated protein 1	0.130693	0.224159	0.402159
Spry1	Sprouty RTK signaling antagonist 1	0.302713	0.171773	0.335768
Fgf18	Fibroblast growth factor 18	0.3377	0.328288	0.519105
Ntf3	Neurotrophin 3	0.612463	0.092376	0.216542
Tgfbi	Transforming growth factor beta induced	2.669462	8.53E-05	0.001498
