# Supplementary material for: MicroRNA‐21a‐5p Promotes Cerebral Angiogenesis in Transient Ischemic Attack by Targeting RBMS3 and Subsequently Modulating the TGFBR1/SMAD2/3 Pathway
Source: CNS Neurosci Ther. 2025 Aug 18;31(8):e70573. doi: 10.1111/cns.70573 (PMC12358734; doi:10.1111/cns.70573)

Full unedited blot for Figure 2B

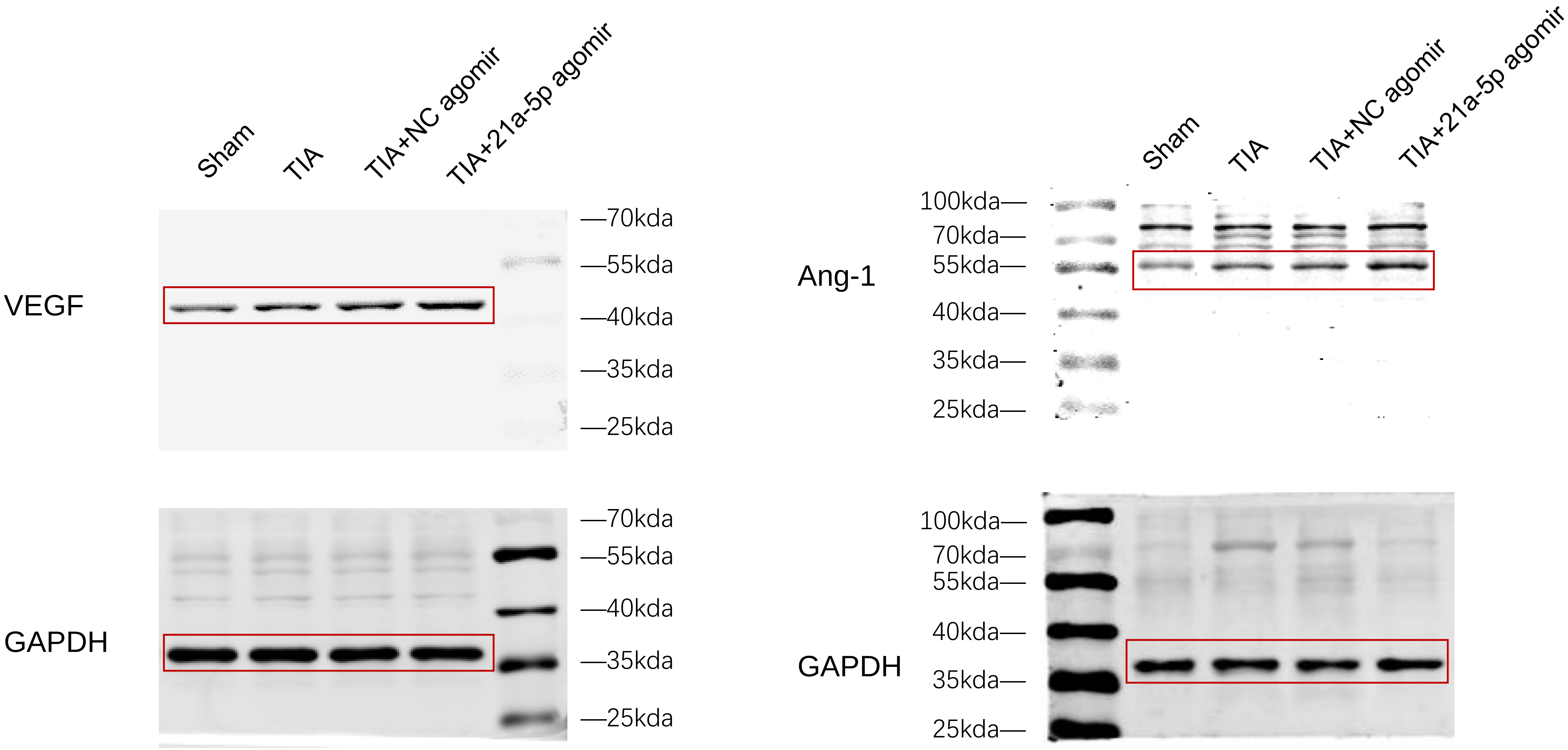

Full unedited blot for Figure 2C

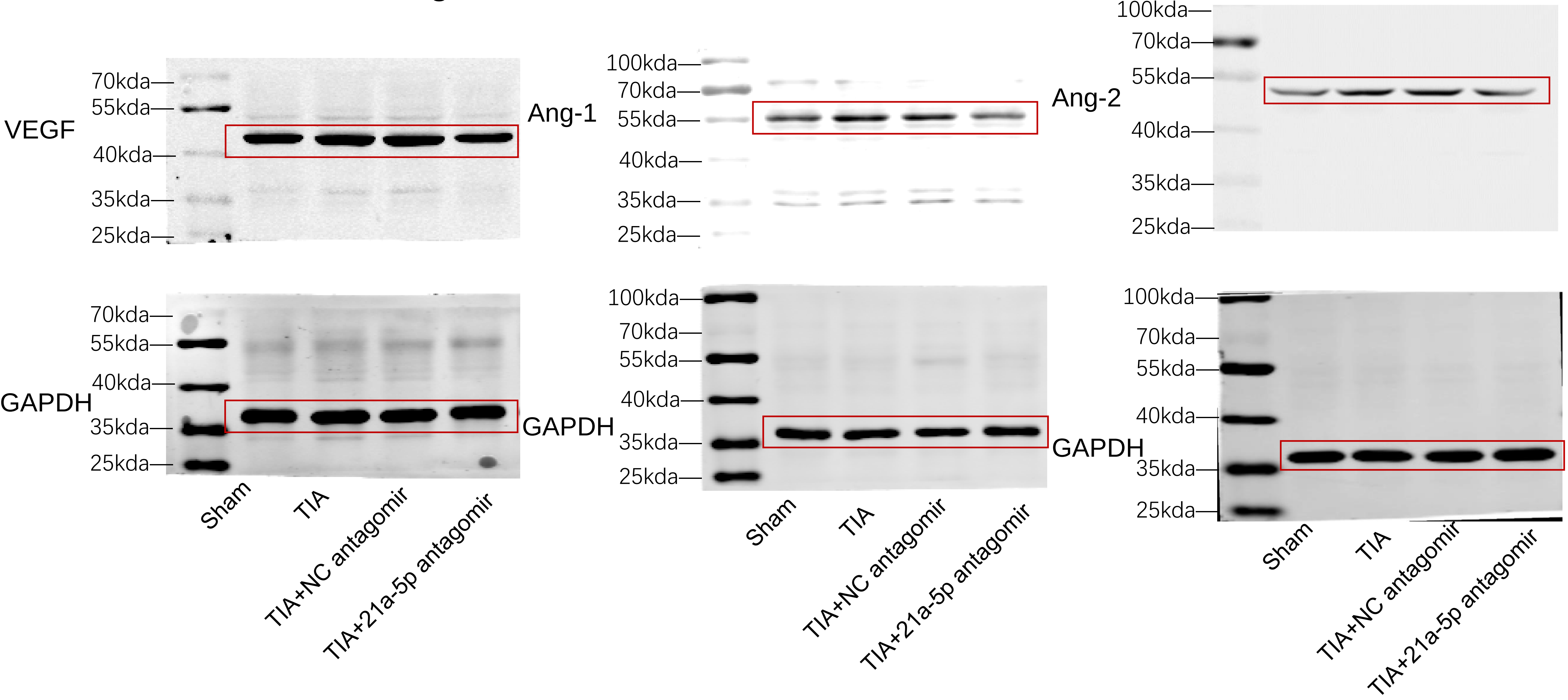

Full unedited blot for Figure 3F

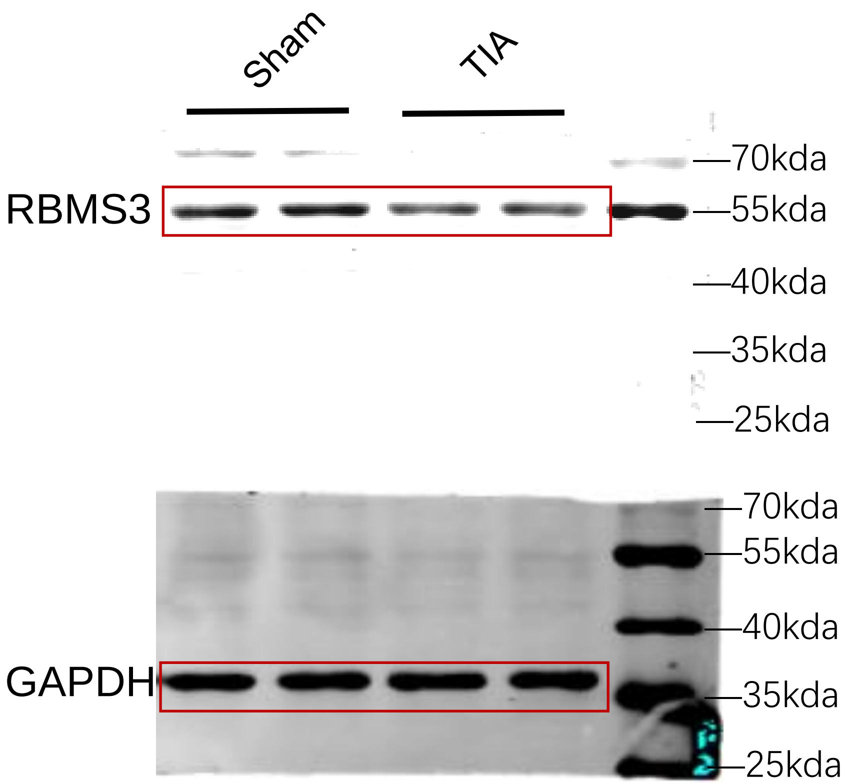

Figure 3I

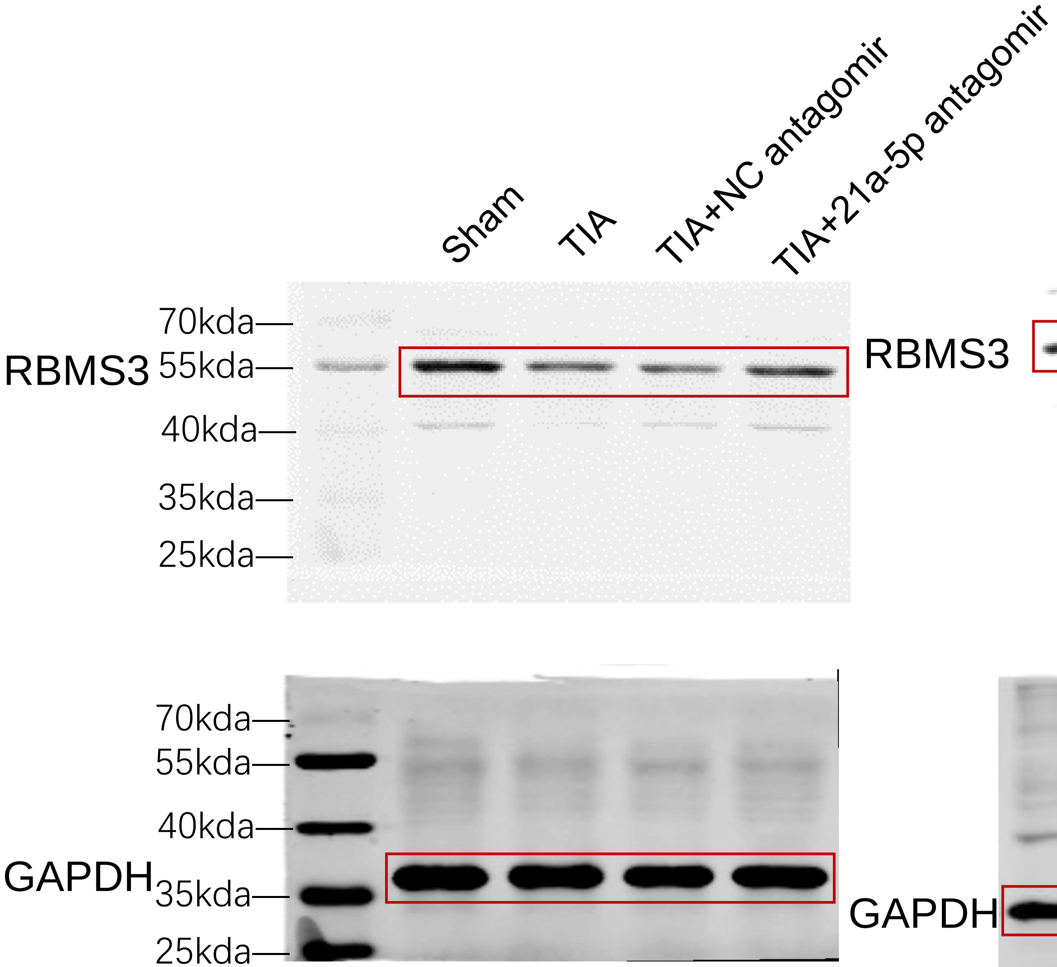

Figure 3K

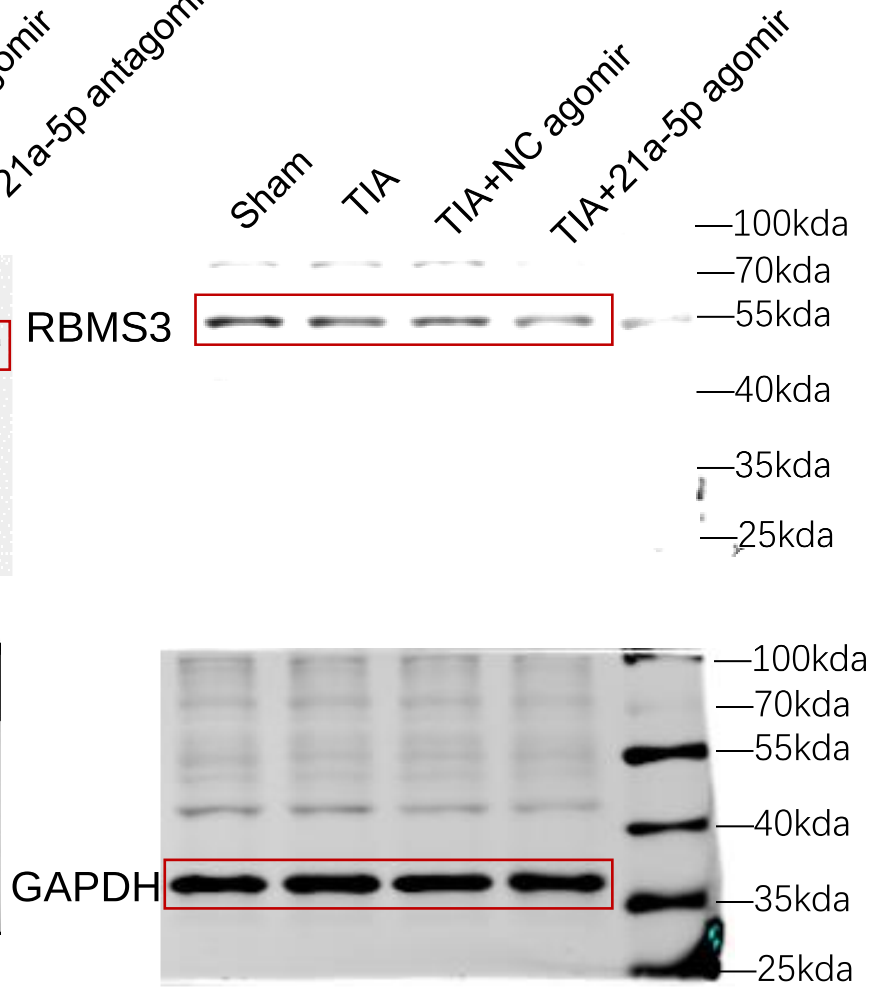

Full unedited blot for Figure 4D

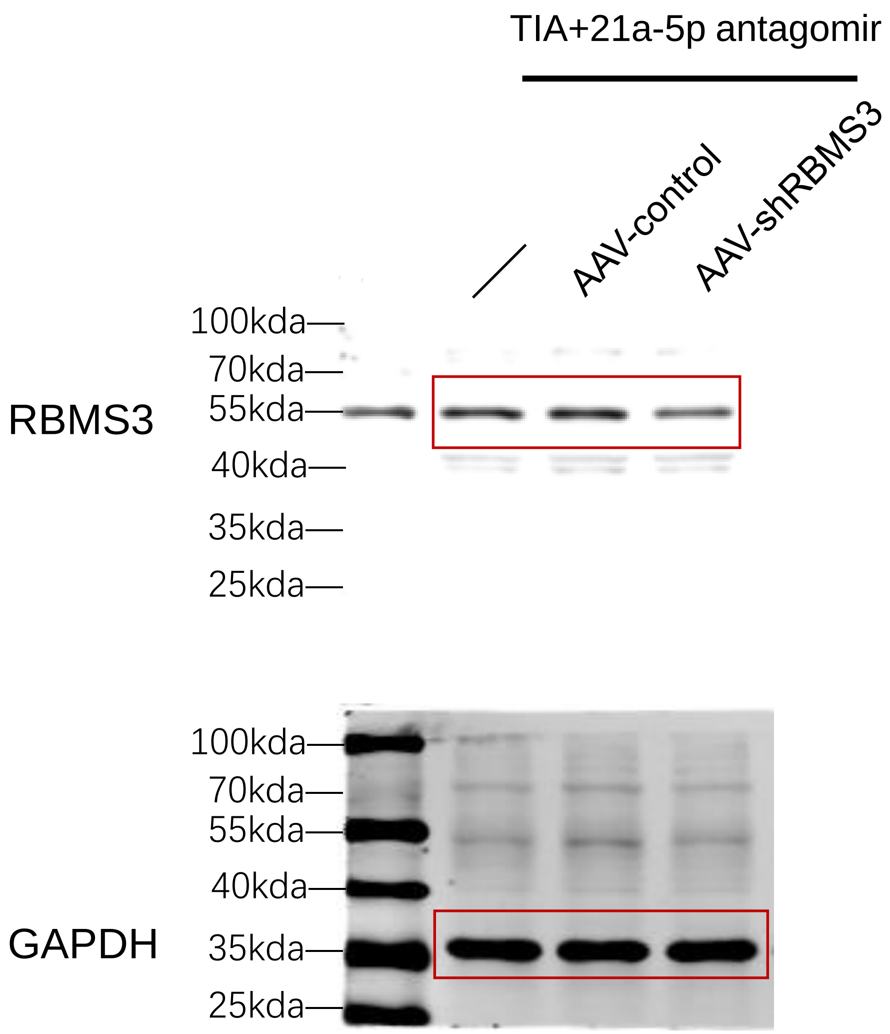

Full unedited blot for Figure 4G

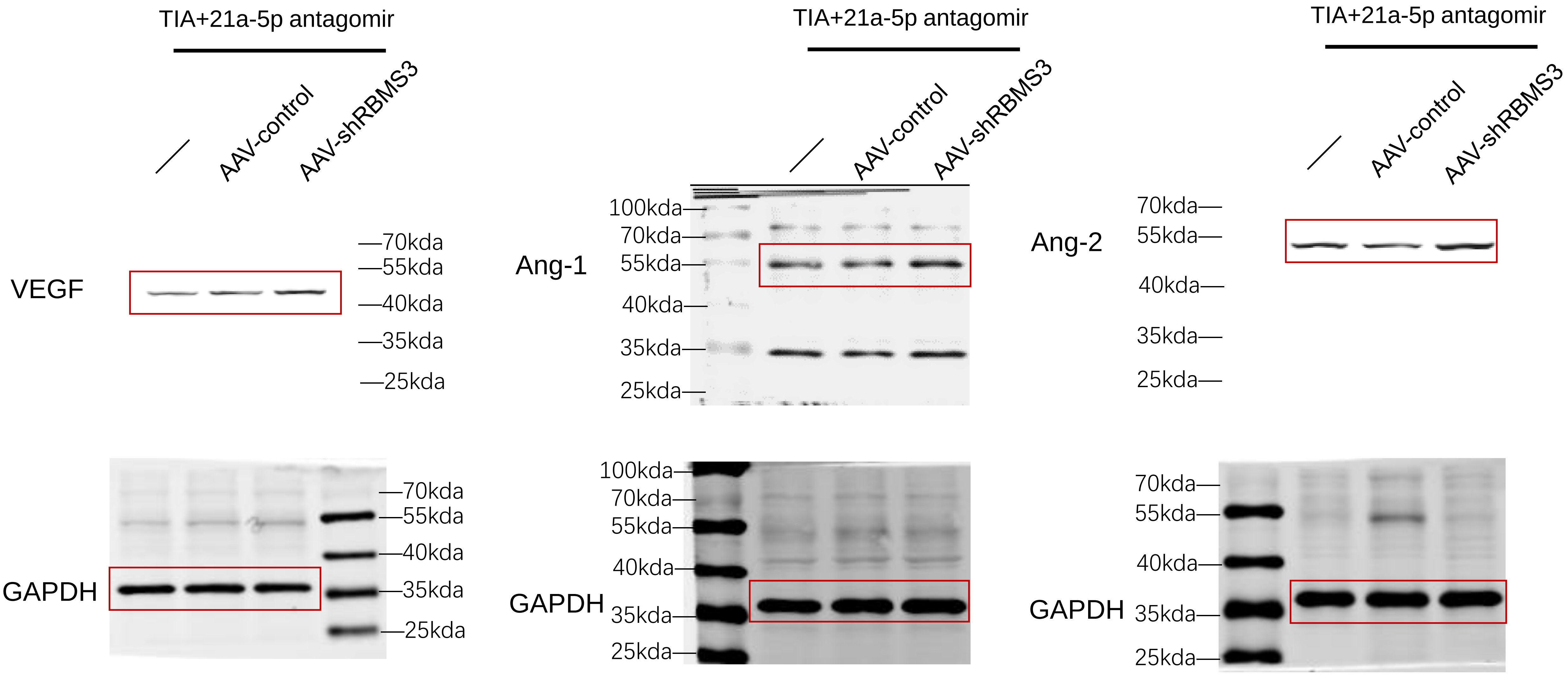

Full unedited blot for Figure 5J

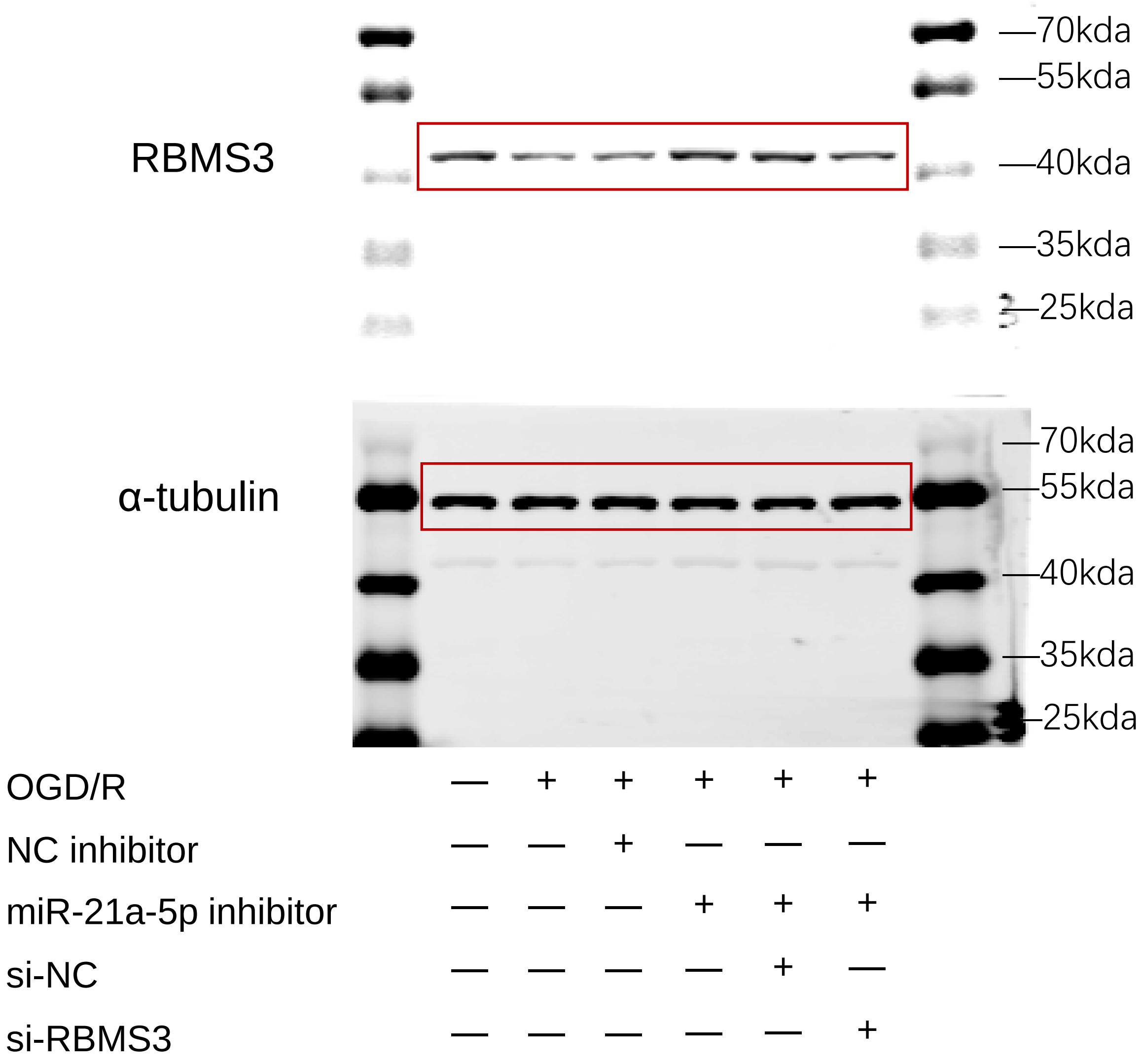

Full unedited blot for Figure 6G

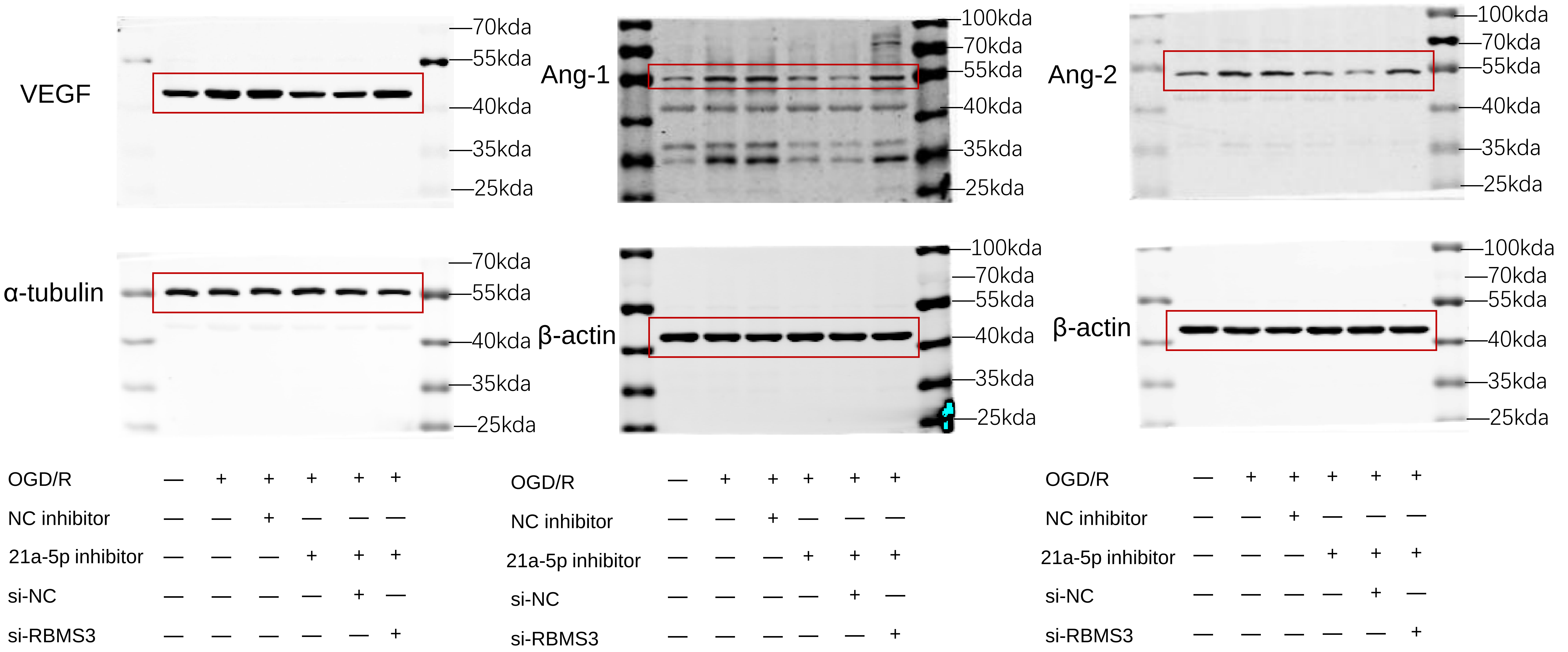

Full unedited blot for Figure 7A

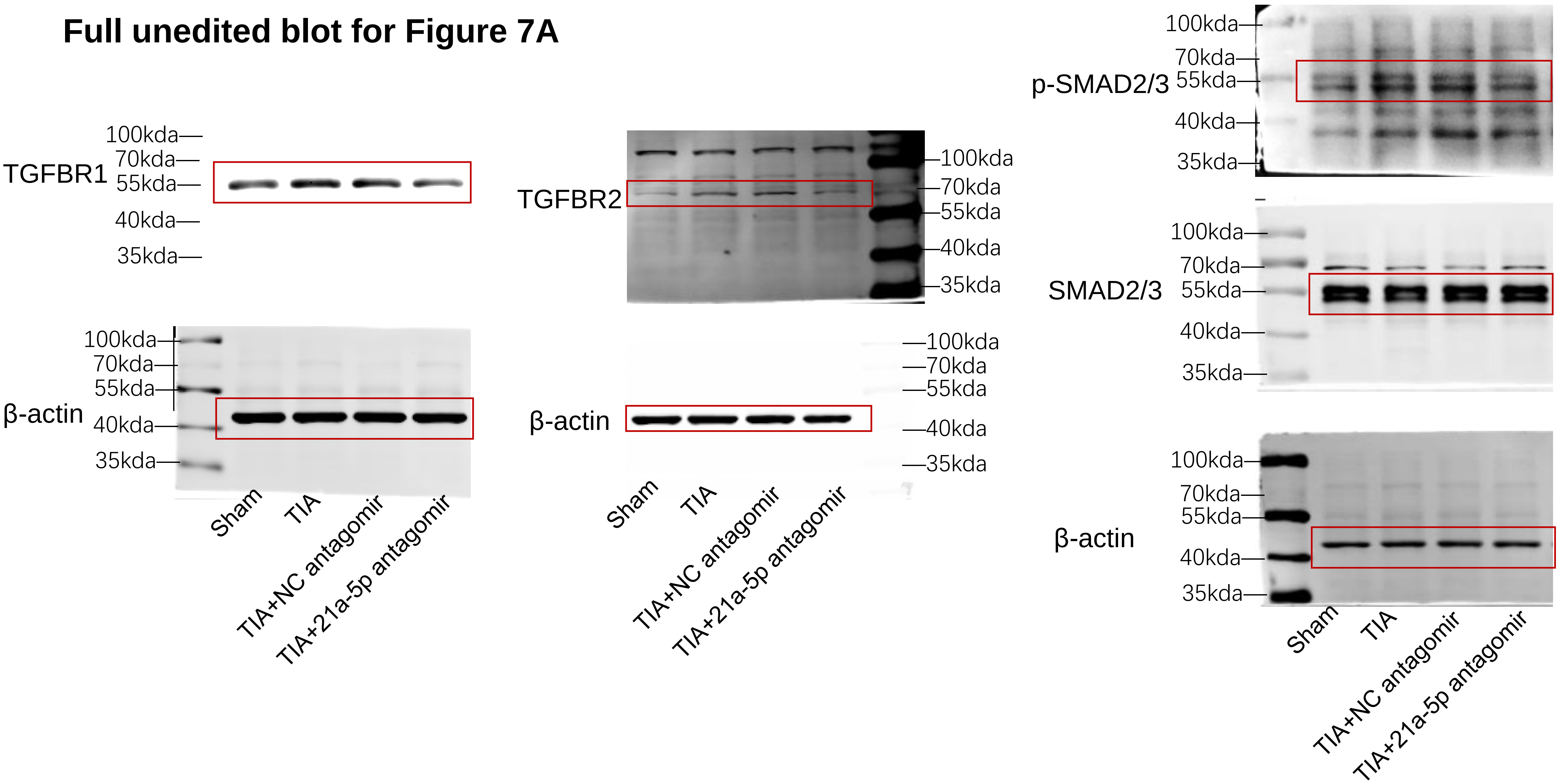

Full unedited blot for Figure 7D

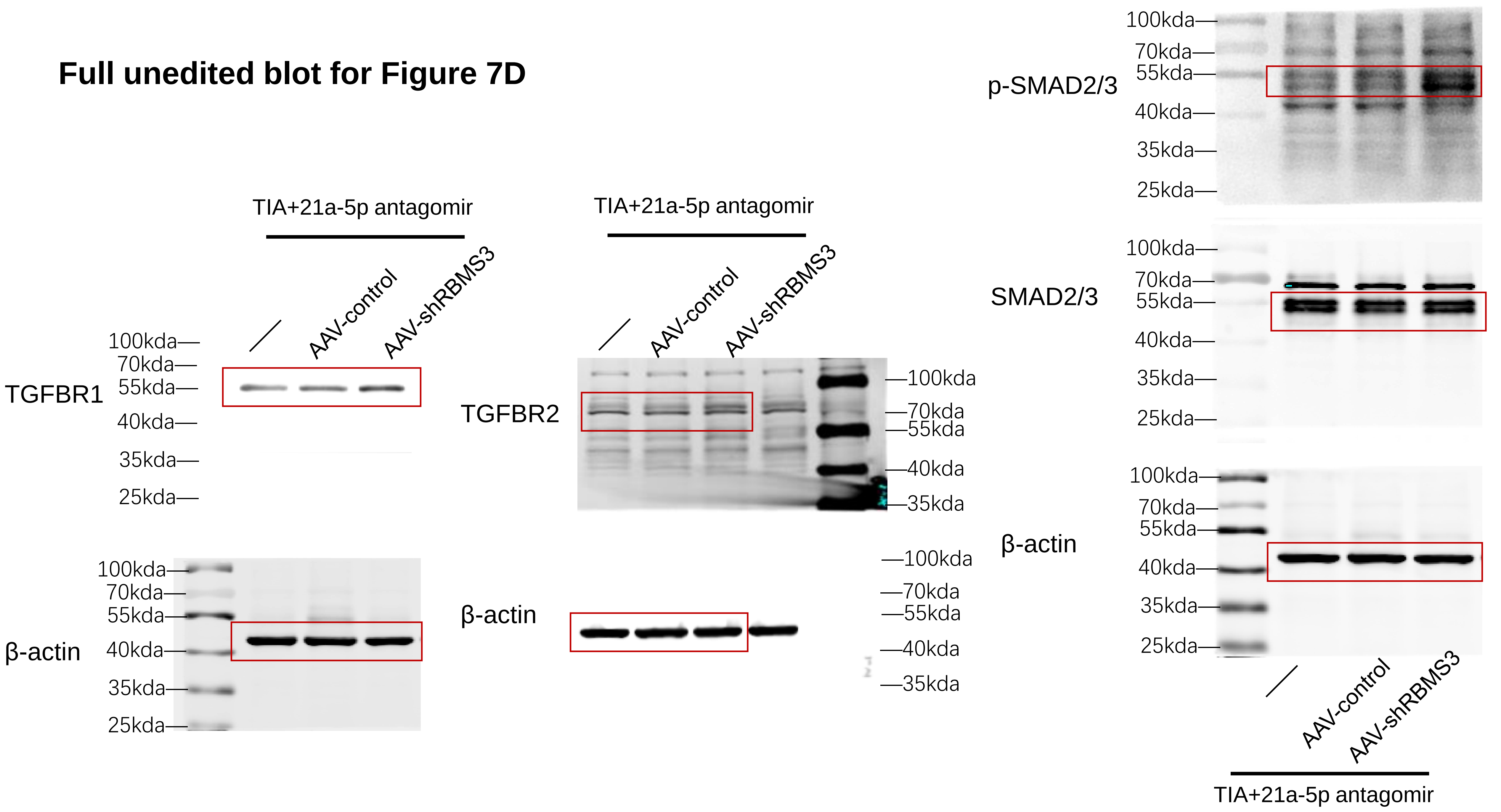

Full unedited blot for Figure 7E

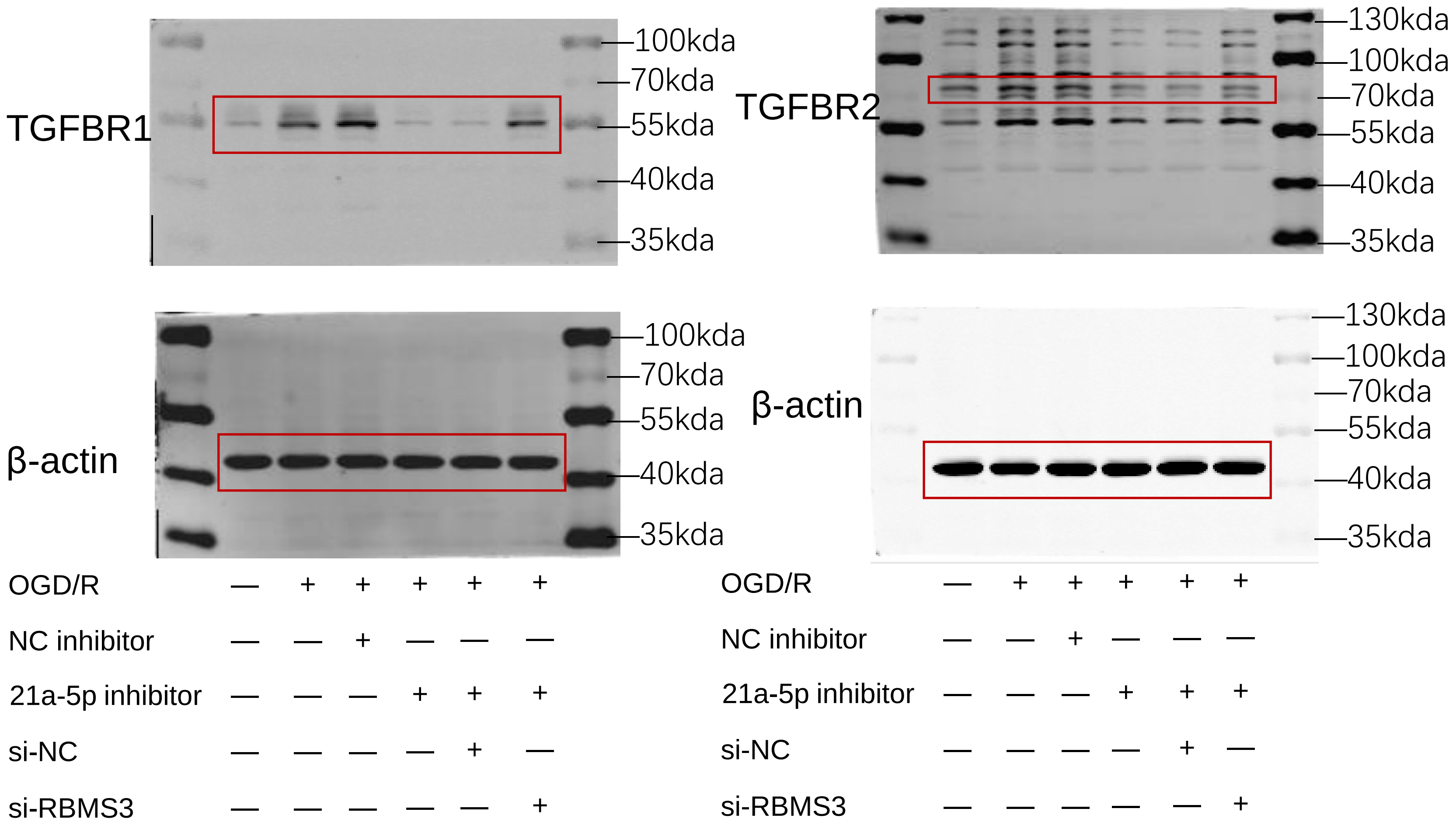

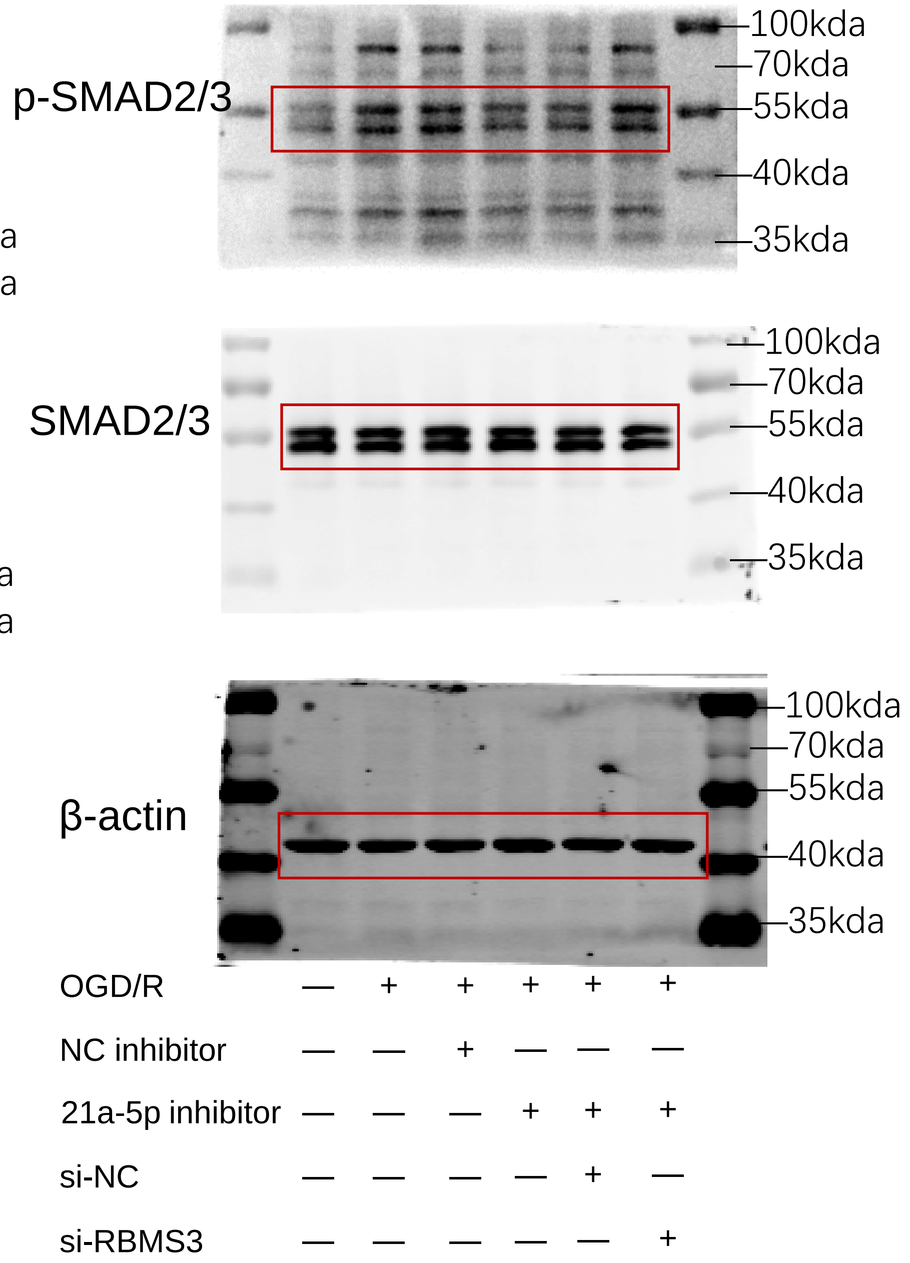

Supplement: Supplementary file 2 — Data S2: cns70573‐sup‐0002‐DataS2.pdf. [file CNS-31-e70573-s001.pdf]
